# The decrease in childhood vaccination coverage and its sociodemographic determinants, the Netherlands, birth cohorts 2008 to 2020

**DOI:** 10.2807/1560-7917.ES.2025.30.39.2500251

**Published:** 2025-10-02

**Authors:** Joyce Pijpers, Annika van Roon, Maarten Schipper, Marijn Stok, Susan van den Hof, Ruben van Gaalen, Susan Hahné, Hester de Melker

**Affiliations:** 1 RIVM, National Institute for Public Health and the Environment, Bilthoven, The Netherlands; 2 Utrecht University, Utrecht, The Netherlands; 3 CBS, Statistics Netherlands, The Hague, The Netherlands; 4 University of Amsterdam, Amsterdam, The Netherlands

**Keywords:** Vaccine-preventable diseases, Vaccination coverage, Measles, Pertussis, Sociodemographic disparities

## Abstract

**INTRODUCTION:**

Childhood vaccination coverage has declined in recent years in many countries, including the Netherlands.

**AIM:**

To understand differences in coverage between population subgroups in the Netherlands over time, we studied sociodemographic factors associated with measles–mumps–rubella (MMR) and diphtheria–tetanus–pertussis–poliomyelitis (DTaP-IPV) vaccination.

**METHODS:**

We conducted a national retrospective database study including children born between 2008 and 2020. Individual-level data linkage allowed examination of associations of sociodemographic variables with MMR and DTaP-IPV vaccination status at age 2 years. We calculated coverage for each variable, stratified by birth cohort. Multivariable Poisson regression assessed independent associations and changes in coverage over time.

**RESULTS:**

MMR coverage decreased in all population subgroups (overall 95% in cohort 2008 and 89% in cohort 2020), more substantially in some. In multivariable analysis, children of non-Dutch origin, particularly Moroccan and Turkish origin, showed more pronounced declines (respectively −25% and −12% as children of Dutch origin in cohort 2020). Among children not attending daycare and children living in larger families (≥ 4 children), coverage declined faster than in those attending daycare and living in smaller families (both −12% in cohort 2020). Coverage among children of self-employed mothers and children in the lowest income households was lower than among children of mothers in employment and the highest income households (respectively −8% and −7% in cohort 2020). Trends for DTaP-IPV vaccination were nearly identical.

**CONCLUSION:**

Childhood vaccination coverage in the Netherlands declined substantially, with increasing disparities between sociodemographic groups. Vaccination efforts should be prioritised to protect public health equitably.

**Key public health message**

**What did you want to address in this study and why?**

Childhood vaccination coverage historically has been high but has decreased in the Netherlands in recent years. Understanding trends across population subgroups over time is important to identify subpopulations at increased risk of vaccine-preventable infections.

**What have we learnt from this study?**

Childhood vaccination coverage has decreased in all population groups in the Netherlands, but the decrease was more substantial in certain groups. A faster decline was observed in Dutch children of non-Dutch origin, children not attending daycare, children of larger families, children of self-employed mothers and those from lower income households.

**What are the implications of your findings for public health?**

The results of our study, reflecting increasing disparities in childhood vaccination coverage, will inform social sciences research investigating underlying reasons for non-uptake, specifically in population groups with lower coverage. In turn, targeted interventions can be developed to provide equitable access to information and vaccination.

## Introduction

Childhood vaccination programmes are the single most effective intervention to protect children’s health. The Expanded Programme on Immunisation is estimated to have averted 154 million deaths globally between 1974 and 2024, the majority in children younger than 5 years [1]. Since 1957, the Netherlands’ National Immunisation Programme (NIP) has provided free, voluntary vaccinations against 13 severe infectious diseases and has significantly reduced child and young adult mortality [2]. Despite its success and historically high coverage, childhood vaccination coverage has declined in recent years in many countries, including in the Netherlands [3]. Coverage for diphtheria–tetanus–pertussis–poliomyelitis vaccination (DTaP-IPV) and measles–mumps–rubella vaccination (MMR) in newborns dropped from 95% in the birth cohort 2008 to 2010 to 93% in cohorts 2015 and 2016 and further to 88% and 89%, respectively, in the 2020 cohort in the Netherlands [4]. Historically, NIP vaccine coverage has been relatively low in Dutch regions where orthodox reformed individuals live (Bible Belt region) [5,6]. However, in recent years a decline in coverage of all childhood vaccinations has occurred also outside of the Bible Belt region, specifically in large cities [4].

(Inter)national research has summarised sociodemographic factors associated with childhood vaccination coverage. Lower (maternal) education level and lower socioeconomic status were associated with lower routine childhood vaccination coverage [7,8]. Similarly, lower parental income, lower parental education level and not being the first-born child were independently associated with lower MMR vaccination uptake [9]. A Dutch study among infants born in 2005 found that parental country of birth and religious objections to vaccination were significant determinants of vaccination uptake [10]. Children with at least one parent born abroad, particularly those with both parents born in Türkiye or Morocco, had 20–30% lower odds of being fully vaccinated [10]. This lower uptake aligns with more recent Dutch studies on HPV and meningococcal (ACWY) vaccination [11-13]. In these studies, data on country of origin were available at individual level, while socioeconomic status (SES) variables and proxy variables for religion (voting proportions for religious political parties) were available at an aggregated level.

The aim of this study was to identify socio-demographic factors associated with changes in vaccination coverage over time, aligning with the ‘situation analysis’ phase of the World Health Organization (WHO) Tailoring Immunization Programmes (TIP) approach, using novel and detailed individual-level data. The WHO TIP model is a structured framework for improving immunisation coverage, consisting of four main phases: situation analysis (review existing data and define target groups), research (gain deeper insight into underlying factors), intervention design (developing targeted strategies) and implementation and evaluation (implementing interventions in real-world settings and systematically evaluating their effectiveness) [14-16]. Insights from this research can inform targeted interventions to improve MMR and DTaP-IPV vaccination coverage, reduce disparities and promote equitable access to vaccination and information.

## Methods

### Study design and population 

For this retrospective cohort study, we linked multiple data sources using a unique identifier to assess determinants of MMR and DTaP-IPV vaccination coverage in the Netherlands of children born between 2008 and 2020. In that period, sociodemographic registries maintained consistent variables and data collection methods. Data linkage was done in the secured remote access environment of Statistics the Netherlands (CBS).

The study population included all children born 2008 to 2020 and was selected from the Personal Records Database (BRP). We used national registries on mortality and migration to determine whether the children had been eligible for routine vaccinations. Specifically, we excluded children who had died or emigrated before becoming eligible for the first dose of MMR (scheduled at age 14 months) or for the DTaP-IPV booster dose (scheduled at age 11 months), as well as children who immigrated to the Netherlands after these respective ages. In addition, we linked children to their parents using the BRP-based register for child–parent relations from Statistics the Netherlands. Children for whom no parent could be identified in the national registries were excluded from the analyses.

### Outcome measure 

The binary outcome measures in this study were uptake before the second birthday of (i) at least one dose of MMR vaccine and (ii) at least three doses of DTaP-IPV vaccine (scheduled at 2 and/or 3 months of age, at 5 months and 11 months), as recommended in the Dutch NIP. An overview of the national vaccination schedule can be found in Supplementary Table S1. We calculated age at time of vaccination based on birth date (month-year) and date of vaccine administration. Vaccination data was available from Praeventis, the register of the Dutch NIP [17]. The vaccination dataset including MMR and DTaP-IPV vaccinations was linked to the study population based on the same unique identifier. Children without a vaccination record in Praeventis were classified as unvaccinated.

### Covariates

We included the following individual-level variables provided by CBS [18] as potential determinants for MMR1 and DTaP-IPV booster vaccination: year of birth, maternal education level (high vs not high (including intermediate, low and unknown), household disposable income, mother’s income source (employed, self-employed, benefit recipient, pension recipient or student), family size (1–3 children, ≥ 4 children or institutional household), urbanisation level, daycare attendance (yes or no), migration status and country of origin. Maternal education level was determined based on the highest completed education. The category ‘high’ education level is relatively well registered, as the registers are older, whereas information on ‘low’ and ‘middle’ education more often is incomplete. Therefore, we dichotomised education as high vs not high. Standardised household disposable income reflects the disposable income, corrected for household size and composition by CBS, and divided into quartiles based on the entire Dutch population. Urbanisation level was defined based on the average address density per square kilometre: extremely urbanised (≥ 2,500 addresses/km^2^), strongly urbanised (1,500–2,499 addresses/km^2^), moderately urbanised (1,000–1,499 addresses/km^2^), hardly urbanised (500–999 addresses/km^2^) and not urbanised (≤ 500 addresses/km^2^). Migration status contained the following categories: Dutch origin (all parents and grandparents born in the Netherlands), migrant or child of at least one first-generation migrant, and child of at least one second-generation migrant. Migrant children and children of first-generation migrants were grouped due to low numbers. Country of origin was categorised as the Netherlands, Europe (excluding the Netherlands), Morocco, Türkiye, Suriname, Dutch Caribbean, Indonesia, Other: America/Oceania, Other: Africa, Other: Asia). Indonesia; Other (Africa, Asia, America/Oceania). Country of origin was primarily defined by the country of birth (CoB) of the child itself. If the child was born in the Netherlands, country of origin was first determined by the parents’ country of birth (CoB). If both parents were born in the Netherlands, the grandparents’ CoB was then considered. Within this definition, the mother’s CoB was prioritised over the father’s, and the grandmother’s CoB was prioritised over the grandfather’s. Foreign-born origins were given precedence over Dutch origin if present in the lineage.

### Statistical analysis

Descriptive analysis summarised the sociodemographic characteristics of the vaccinated and unvaccinated children. Vaccination coverage was calculated as the number of children vaccinated before their second birthday divided by the total population eligible for each category of the sociodemographic variables.

We used a multivariable Poisson regression model to assess the independent association between vaccination and the sociodemographic variables. The results are reported as adjusted rate ratios (RRs) with 95% confidence interval levels (CI) and p values. The level of significance was set at 5%. Since the proportion of missing categorical values was small (≤ 3%), they were excluded from the multivariable regression analysis. To account for potential nonlinearity of the trend of vaccination coverage over time, we included the variable birth cohort in the model as a categorical variable. The variable migration status was excluded from the multivariable analysis, given its complete overlap with the variable country of origin. To assess changes in the associations over time, we included interaction terms between the variable birth cohort and each determinant in the multivariable model. We report the adjusted RRs (aRR) for each category within the sociodemographic variables per birth cohort and subsequently, we calculated the adjusted relative change in vaccination coverage ((aRR – 1) × 100), based on the aRR of the total effect (main effect birth year + main effect socio-demographic variable + interaction effect). We calculated the number of unvaccinated children in each category for the most recent birth cohort to pinpoint where interventions could have the greatest impact on vaccination coverage; these calculations are appended in Supplementary Table S7.

All analyses were performed in R version 4.4.0 [19]. This manuscript was prepared in accordance with the Strengthening the Reporting of Observational Studies in Epidemiology (STROBE) guidelines [20].

## Results

### Descriptive analysis

The total population of the 13 included birth cohorts for MMR and DTaP-IPV included 2,323,838 and 2,331,199 children, respectively (Table). Overall, by the age of 2 years, 2,174,229 children (94%) were vaccinated for MMR, and 2,172,402 children (93%) were vaccinated for DTaP-IPV. The distribution of sociodemographic groups in both the MMR and DTaP-IPV population are described in Table. Of children eligible for both MMR and DTaP-IPV vaccination (n = 2,319,001), 97.2% had the same vaccination status for both vaccines (either vaccinated or unvaccinated), 1.4% only received the MMR vaccination and 1.3% only received the DTaP-IVP vaccination. Supplementary Table S2 additionally provides the distribution of the sociodemographic variables per birth cohort.

**Table:** MMR and DTaP-IPV vaccination uptake at age 2 years, by sociodemographic characteristics, the Netherlands, children born 2008–2020 (n = 2,323,838 for MMR; n = 2,331,199 for DTaP-IPV)

	MMR				DTaP-IPV			
	Population		Vaccinated		Population		Vaccinated	
	n	Column %	n	Row %	n	Column %	n	Row %
Education level mother								
High	879,390	38	841,000	96	878,790	38	842,050	96
Not high^a^	1,444,450	62	1,333,230	92	1,452,410	62	1,330,350	92
• Intermediate	708,950	31	663,840	94	708,600	30	666,340	94
• Low	256,880	11	241,910	94	256,550	11	241,980	94
• Unknown	478,620	21	427,480	89	487,270	21	422,030	87
Household standardised disposable income ^b^								
First quartile	316,700	14	289,380	91	315,930	14	288,750	91
Second quartile	645,820	28	602,760	93	645,450	28	603,710	94
Third quartile	758,400	33	725,150	96	757,760	33	725,960	96
Fourth quartile	535,960	23	515,700	96	535,130	23	514,710	96
Unknown	66,970	3	41,240	62	76,930	3	39,280	51
Income source mother								
Job in employment	1,554,150	67	1,494,390	96	1,552,980	67	1,495,390	96
Self-employed	178,250	8	161,340	91	178,090	8	161,780	91
Benefit recipient	273,210	12	253,600	93	272,400	12	253,710	93
Pension recipient	10,580	< 1	9,830	93	10,560	< 1	9,840	93
Student	35,430	2	33,050	93	35,320	2	33,060	94
Other	205,780	9	181,710	88	205,060	9	180,020	88
Unknown	66,450	3	40,310	61	76,800	3	38,600	50
Migration status								
Dutch origin	1,407,980	61	1,337,740	95	1,385,260	59	1,318,650	95
Migrant or child of first-generation migrant(s)	648,100	28	586,510	90	654,160	28	580,040	89
Child of second-generation migrant(s)	267,760	12	249,990	93	291,790	13	273,710	94
Country or origin^c^								
The Netherlands	1,407,970	61	1,337,740	95	1,385,260	59	1,318,660	95
Europe (excl, the Netherlands)	266,380	11	237,350	89	276,730	12	239,190	86
Morocco	104,630	5	93,230	89	107,290	5	96,160	90
Türkiye	85,260	4	79,480	93	88,040	4	82,300	93
Suriname	71,860	3	67,940	95	75,310	3	71,160	94
The Dutch Caribbean	45,030	2	41,480	92	46,460	2	42,930	92
Indonesia	74,320	3	70,960	95	78,880	3	75,510	96
Other, America/Oceania	61,340	3	55,770	91	63,430	3	55,600	88
Other, Africa	78,060	3	72,120	92	79,180	3	73,170	92
Other, Asia	129,000	6	118,170	92	130,630	6	117,730	90
Level of urbanisation								
Extremely urbanised	501,210	22	471,400	94	500,170	21	470,200	94
Strongly urbanised	579,000	25	550,330	95	578,190	25	550,390	95
Moderately urbanised	440,560	19	419,740	95	440,110	19	419,760	95
Hardly urbanised	396,580	17	375,890	95	396,290	17	376,180	95
Not urbanised	344,940	15	320,720	93	344,840	15	321,030	93
Unknown	61,540	3	36,150	59	71,610	3	34,840	49
Family size								
1–3 children	2,119,770	91	2,017,780	95	2,117,000	91	2,016,800	95
≥ 4 children	138,590	6	117,210	85	138,410	6	117,520	85
Institutional household	4,260	< 1	3,360	79	4,260	< 1	3,310	78
Unknown	61,220	3	35,880	59	71,530	3	34,780	49
Daycare attendance								
Yes	1,389,670	60	1,342,800	97	1,388,140	60	1,343,320	97
No	934,030	40	831,290	89	942,910	40	828,940	88
Unknown	139	<1	137	<1	148	<1	141	<1

MMR: Measles–mumps–rubella; DTaP-IPV: diphtheria, tetanus, pertussis, poliomyelitis.

^a^ The category ‘not high’ included intermediate, low and unknown education level.

^b^ If parents of children were living in separate households, the average SDI of the two households was taken. Quartiles (percentiles) were calculated by Statistics the Netherlands based on the complete Dutch population.

^c^ The country of origin was determined based on the country of birth of the child included in the study, and the countries of birth of its parents and grandparents.

All tables exclude frequencies < 10 and all numbers are rounded to the nearest ten, to avoid personally identifiable information. Therefore, the totals in the table may differ slightly from the total number of children in the study population.

### Sociodemographic factors associated with vaccination coverage over time

**Figure 1:**
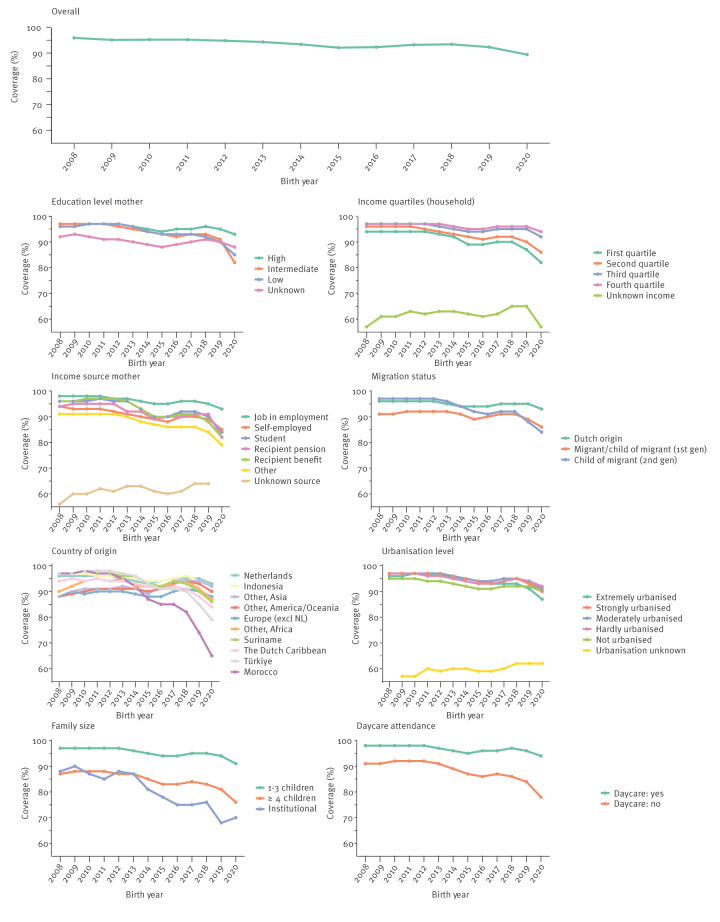
Unadjusted measles–mumps–rubella vaccination coverage at age 2 years, per birth cohort, the Netherlands, children born 2008–2020 (n = 2,323,838)

Overall, crude MMR vaccination coverage declined from 95% in cohort 2008 to 89% in cohort 2020, and a decrease was observed across all sociodemographic groups (Figure 1).

gen: generation; NL: the Netherlands.

The vaccination coverage for cohort 2020 may be underestimated due to informed consent.

The MMR vaccination coverage was not associated with maternal educational level in 2008 (97%), but this changed over time: children of mothers who had higher education levels maintained relatively high coverage over time (94% in cohort 2020) while it decreased to 85% in cohort 2020 for children of mothers with low educational level. Lower vaccination coverage was consistently present among children in the lowest (first) income quartile households (94% in cohort 2008 and 82% in cohort 2020) and those with self-employed mothers (94% in cohort 2008 and 85% in cohort 2020). In the 2008 cohort, children of second-generation migrants (at least one grandparent born abroad) had higher coverage compared with the combined group of migrant children (born abroad themselves) and children of first-generation migrant (at least one parent born abroad), 97% and 81%, respectively. Coverage declined faster over time in children of second-generation migrants, so that in cohort 2020, coverage (84%) was lower than the combined group of migrant children and children of first-generation migrants (86%). Children of Dutch origin had relatively high vaccination coverage (96% in cohorts 2008 to 2012 and 93% in in cohort 2020) compared with Dutch children of non-Dutch origin. Specifically Dutch children of Moroccan and Turkish origin had a 1–2% higher coverage in birth cohorts 2008 to 2012 compared with children of Dutch origin, but experienced a steeper decline in coverage from cohort 2014 onwards (from 92% in cohort 2014 to 65% in cohort 2020 and from 96% in cohort 2014 to 79% in cohort 2020, for Dutch children of, respectively, Moroccan and Turkish origin). Within the group of Dutch children of Moroccan origin, coverage since cohort 2013 has been lower for children of second-generation migrants compared with the combined group of migrant children and children of first-generation migrants (58% and 71% in cohort 2020, respectively). Within the group of Dutch children of Turkish origin, coverage was only in cohort 2020 slightly lower for children of second-generation migrants compared with the combined group of migrant children and children of first-generation migrants (84% and 86%, respectively). Geographical factors also played a role, with children living in non-urbanised areas consistently showing lowest coverage until cohort 2019, whereafter coverage of children in extremely urbanised areas was lowest (87% in cohort 2020). Moreover, coverage was consistently lower in children living in large families (76% in cohort 2020), children living in institutional households (70% in cohort 2020) and children not attending daycare (78% in cohort 2020).

DTaP-IPV vaccination coverage decreased from 95% in cohort 2008 to 90% in cohort 2020. The DTaP-IPV coverage by country of origin showed some slight differences compared with MMR coverage, with Dutch children from European origin (excluding the Netherlands), ‘Other, America/Oceania’ and ‘Other, Asia’ having lower coverage in earlier cohorts (2008–2014). Aside from this, similar trends were observed in the crude DTaP-IPV vaccination coverage over time; for a detailed overview of DTaP-IPV coverage by sociodemographic variables see Supplementary Figure S3.

### Sociodemographic factors associated with vaccination coverage

**Figure 2:**
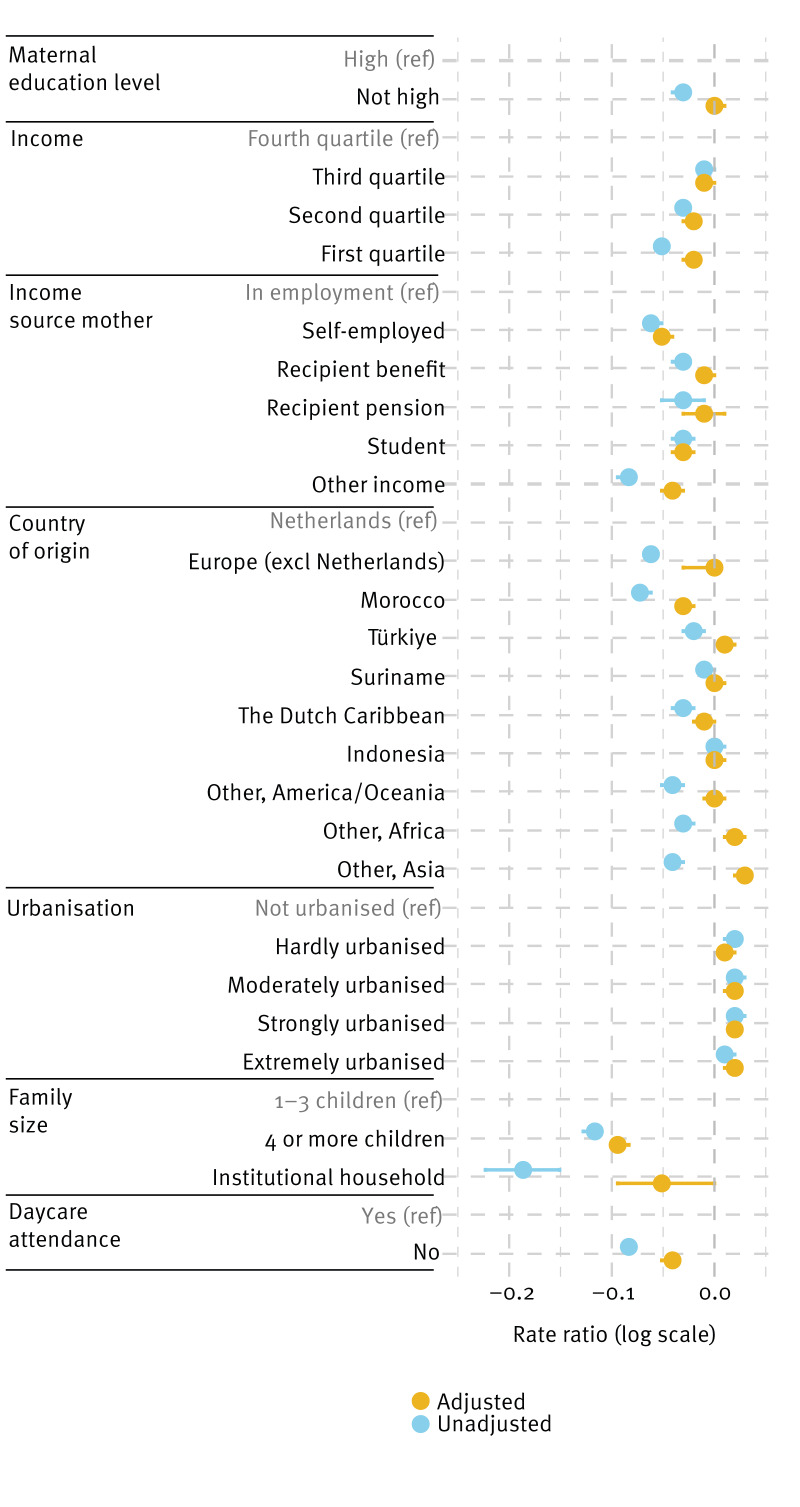
Unadjusted and adjusted rate ratios for measles–mumps–rubella vaccination coverage by sociodemographic factors, the Netherlands, children born 2008–2020 (n = 2,242,990)

Figure 2 shows the univariable and multivariable regression analysis for MMR vaccination and corresponding RR (unadjusted and adjusted). The figure shows variations in the RR across different sociodemographic groups. Overall, the strongest associations with lower vaccination coverage in adjusted analyses were related to larger family size (≥ 4 children; aRR = 0.91), living in an institutional household (aRR = 0.95) and not-attending daycare (aRR = 0.96). Maternal education level overall showed a nuanced effect: while lower education was associated with lower vaccination coverage in the unadjusted model (RR = 0.97), the adjusted model indicated minimal difference between children of mothers with high education level and children of mothers without high education level (aRR = 1.00). With regard to the economic variables: lower standardised disposable income of the household was associated with lower vaccination coverage in the adjusted model, particularly in the first quartile (aRR = 0.98) as was having a self-employed mother or a mother with ‘other’ income (aRR = 0.95 or aRR = 0.96, respectively). The association between country of origin and vaccine coverage varied: Dutch children of Moroccan origin had lower coverage than children of Dutch origin (aRR = 0.97), while Dutch children of Turkish, African and Asian origin showed higher coverage than children of Dutch origin (aRR = 1.01 for Türkiye, aRR = 1.02 for Africa and aRR = 1.03 for Asia). Living in hardly to extremely urbanised areas, compared with non-urbanised areas, was positively associated with MMR vaccination coverage (aRR = 1.02 for strongly urbanised areas).

ref: reference.

In addition to all the variables listed, the RRs are also adjusted for birth cohort.

With regard to DTaP-IPV vaccination coverage, we found similar results; these are appended in Supplementary Figure S4.

### Changes in sociodemographic factors associated with vaccination coverage over time

**Figure 3:**
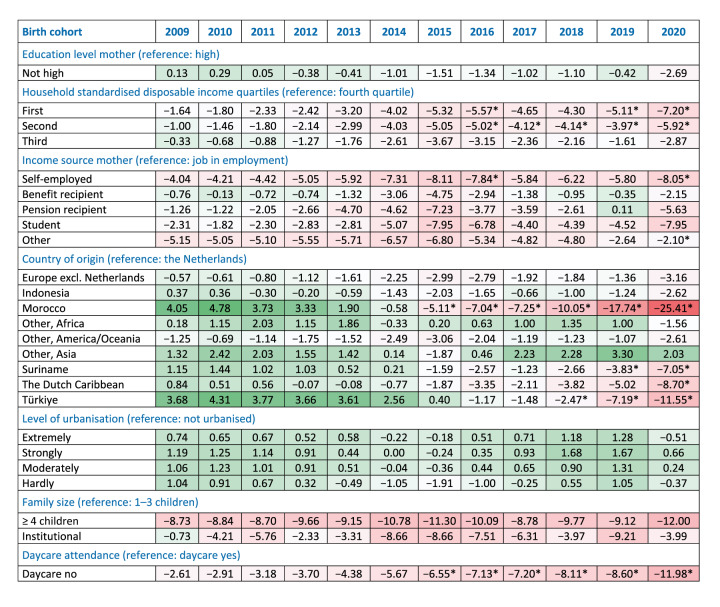
Adjusted relative change in measles–mumps–rubella vaccination coverage compared with the reference cohort 2008 for each stratum of the covariates, based on the rate ratios derived from the multivariable regression model with interaction terms for birth cohort, the Netherlands, children born 2008–2020 (n = 2,242,990)

The multivariable regression model including interaction terms for birth cohort revealed significant variations in MMR vaccination coverage over time; the extensive model can be found in Supplementary Table S5. Figure 3 presents the relative changes in vaccination coverage across sociodemographic groups relative to cohort 2008, calculated based on the estimates from the model and adjusted for baseline coverage and all sociodemographic variables. Country of origin played a significant role, with Dutch children of Moroccan origin having the most pronounced decline in coverage compared with Dutch children of Dutch origin, starting from a 4% higher coverage in cohort 2009 to a 25% lower coverage in cohort 2020. Dutch children of Turkish origin also showed a substantial decrease in coverage, with a 4% higher coverage in cohort 2009 and a 12% lower coverage in cohort 2020, compared with Dutch children of Dutch origin. Other notable declines were observed among Dutch children of Dutch Caribbean origin (−9% in cohort 2020) and Dutch children of Surinamese origin (−7% in cohort 2020). Daycare attendance and family size were also significantly associated with differential changes in vaccination coverage. Children not attending daycare had increasingly lower coverage compared with children attending daycare (−3% in cohort 2009 and −12% in cohort 2020). No significant differential changes in vaccination coverage over time were found for maternal educational level, urbanisation level and household size. Children living in families with four or more children, however, consistently had a lower vaccination coverage than children living in smaller families, with the largest difference in cohort 2020 (−12%). However, these differences have remained stable over time. Socioeconomic disparities were observed and became slightly wider in cohort 2020, with consistently lower coverage among children of self-employed mothers compared with children of mothers with a job in employment (−8% in cohort 2020) and lower coverage among children in the lowest income households compared with the highest income households (−7% in cohort 2020).

* Significant values (p < 0.05).

We append in Supplementary Table S5 a sensitivity analysis including missing values, which showed minimal changes in the model estimates. Regarding DTaP-IPV vaccination, similar trends were observed; the multivariable model for this can be found in Supplementary Table S4 and the adjusted relative changes in DTaP-IPV vaccination over time in Supplementary Table S6.

## Discussion

Our study revealed a substantial decline in MMR and DTaP-IPV vaccination coverage in the Netherlands from birth cohort 2008 to 2020, with a more pronounced and faster decline observed in specific sociodemographic subgroups. This trend increases the risk for outbreaks of vaccine preventable diseases and indicates rising inequity in vaccination coverage. This study presents an application of the situation analysis phase of the WHO TIP framework [14-16]. Further research within the SocioVax programme will explore barriers and drivers of vaccination in subgroups with lower coverage [21].

The general decline in vaccination coverage in the Netherlands is consistent with trends reported in other countries [3,22]. The coverage for the first dose of measles-containing vaccine in the European Union and European Economic Area decreased from 95% in 2018 to 92% in 2022 [23]. Widening inequities in childhood vaccination have also been reported in the United Kingdom, particularly with regards to ethnicity and deprivation level [24-26].

General explanations for the decline in vaccination coverage include distrust in vaccines and governmental organisations, misinformation, and the influence of social media [27-29]. Studies link reduced vaccination uptake to scepticism towards governmental policies and lack of confidence in vaccines [29]. The COVID-19 pandemic may have exacerbated these issues, and targeted strategies may be required to promote vaccination uptake in the post-pandemic era [30,31]. Trust in governmental institutions among people in the Netherlands in 2022 was below the pre-pandemic level, especially among individuals with a lower education level, and was associated with lower COVID-19 vaccine uptake [32]. Parental perceptions about childhood vaccination also became slightly more negative after the pandemic [33], although coverage had already been declining earlier. Social media also play a role in the spread of misinformation. Research on Dutch Twitter activity in 2019 showed how anti-vaccine narratives gained attention and influenced public opinion [28]. Because of selective exposure, individuals with more sceptical attitudes towards vaccination are more likely to encounter negative messages about vaccination, reinforcing their views [28]. The anti-vaccination message on the Internet is much less restrained than on other media, which makes it more likely that the public takes uninformed decisions about vaccination [27].

Crucially, our findings indicate that specific sociodemographic groups experience a more pronounced and faster decrease in vaccination coverage. The most substantial relative declines were observed among Dutch children of non-Dutch origin, children not attending daycare, those with self-employed mothers and children in the lowest income households. Vaccination coverage declined notably among Dutch children with a migration background, specifically children of Moroccan, Turkish, Dutch-Caribbean and Surinamese origin, also after adjusting for other variables. These findings are consistent with earlier studies in the Netherlands on NIP coverage at national and municipal levels [10,13]. The potential social clustering of unvaccinated children within these populations poses public health risks by increasing outbreak potential [34]. Several qualitative studies have been conducted to provide further insights in reasons behind lower vaccination coverage among Dutch children with a migration background. The results of a focus group study among parents of Moroccan and Turkish origin in 2015 indicated difficulty in understanding information about the NIP and in reaching vaccination locations (i.e. child welfare centres) as important practical barriers to vaccination [35]. In the same study, some parents mentioned that limited consultation time restricted their opportunity to ask questions about vaccinations [35]. Other reasons may be equally or more important given that Morocco currently is experiencing a large measles epidemic, following declining vaccination rates after the COVID-19 pandemic [36]. Unlike objections in the Orthodox Reformed population [5], no clear Islamic religious objection to vaccination has been identified [35,37,38]. While some studies mention violation of dietary laws (e.g. non-halal-based vaccines), we found no difference between MMR (where one of the two widely used vaccine contains porcine gelatine and is therefore non-halal) and DTaP-IPV (halal vaccines) coverage.

Moreover, children not attending daycare showed lower vaccination coverage. A Canadian systematic review found varying associations between daycare attendance and infant vaccination uptake [39]. In the Dutch context, even though vaccination is not required for daycare attendance, parents are often asked about their child’s vaccination status, which may influence their vaccination decision. As daycare attendance is likely to be associated with other sociodemographic variables, there may be residual confounding after adjusting for available information. Because of close contacts among children, daycare centres can be significant hotspots for infectious disease transmission, thus the higher vaccination coverage in these settings is advantageous for preventing outbreaks.

Children living in larger families (≥ 4 children) had lower vaccination coverage than those in smaller families. This may partly be explained by the higher prevalence of large households within the Orthodox Protestant communities, where vaccine hesitancy is more common [5,40]. Furthermore, our data showed larger families are more common in Dutch children of Moroccan origin and in lower income groups, both associated with lower vaccine coverage. Statistics the Netherlands reports Dutch women of Moroccan origin have, on average, two children, compared with 1.5 among Dutch women of Dutch origin [41]. Although lower income was historically associated with larger family size, recent trends suggest higher income is becoming an increasingly important factor for having more children, specifically for mothers [42].

The socioeconomic disparities observed in our study are consistent with findings from previous research. Systematic reviews reported persisting lower routine childhood vaccination coverage among children with lower parental socioeconomic status [43,44]. Contrary to low- and middle-income countries where (financial) access is often a barrier to vaccine uptake in lower socioeconomic groups, economic disparities in high-income countries may be more related to issues of perceived risks (both of the diseases against which vaccines protect as well as of the vaccines themselves), trust in the government and in healthcare professionals, and vaccine confidence [43,44]. However, to address these disparities, the effects of factors on vaccine uptake among people from lower socioeconomic positions need further research to investigate underlying causes.

While we described some findings about underlying factors from research in the general population it is unclear whether, and to what extent, these same factors play a role in the vaccination uptake of people from these specific subgroups. To address this, future studies within the SocioVax programme will involve members of target groups in the design and implementation phases to jointly develop research that investigates barriers and drivers of vaccination in subgroups with lower coverage [21].

Low coverage becomes a notable problem especially when unvaccinated individuals cluster. For example, children with similar migration backgrounds may cluster within households, families, schools and geographically (e.g. larger cities). This social clustering potentially creates environments where infections spread more easily. Therefore, further research to investigate networks of unvaccinated and nonimmune individuals is needed to assess the risk of spread of vaccine-preventable diseases.

A strength of our study is the use of nationwide registry data and the possibility to link vaccination data at individual level, avoiding ecological fallacies that may result from aggregated data. The results provide a comprehensive understanding of factors influencing changes in vaccination coverage and help identify potential risk groups for disease outbreaks. A limitation is the requirement for informed consent for registration in the national immunisation register for a subset of children born in 2020. Children who could not be linked to the vaccination database were categorised as unvaccinated. This may be differential between subgroups (data on characteristics of parents not providing consent are not available). However, the effect is limited. For MMR vaccination, only children born in November and December (approximately one sixth of the 2020 cohort) were scheduled for vaccination after the introduction of the informed consent in January 2022. For the DTaP-IPV vaccination, all children born in 2020 were scheduled for vaccination before the introduction of the informed consent. The percentage of parents not providing informed consent for vaccination was 3.1% for DTaP-IPV and 4.5% for MMR in 2022 [45]. Another limitation is that children with unknown sociodemographic information, often born abroad, had much lower registered vaccination coverage, possibly due to unrecorded vaccinations received outside the Netherlands. However, missing values were below 3%, and a sensitivity analysis including these unknown categories showed no impact on the results. Finally, the 2019 and 2020 cohorts were the first to be vaccinated during the COVID-19 pandemic, which may have caused delays. Nonetheless, when vaccination status was assessed at age 3 years instead of 2 years, overall coverage for these cohorts remained unchanged. Another limitation of this study is that our findings are based on the Dutch NIP and national context, which may limit their direct applicability to other countries. Nevertheless, as countries are facing similar challenges with declining vaccination coverage and persisting health inequalities, our analytical approach and the sociodemographic determinants identified in this may offer valuable insights.

## Conclusion

We found a substantial decline in MMR and DTaP-IPV childhood vaccination coverage in the Netherlands, with increasing disparities between sociodemographic strata. The potential social clustering of these unvaccinated children increases the risk of localised outbreaks. Future research should focus on understanding the underlying reasons for non-uptake of childhood vaccinations, focusing on populations with lower uptake, according to the WHO TIP framework. Social science research can inform interventions with the aim to increase childhood vaccination coverage.

## Data Availability

All data are available within CBS Microdata and can be made available under strict conditions.

## Supplementary Data

1506000 Supplement
