# Supplementary material for: The decrease in childhood vaccination coverage and its sociodemographic determinants, the Netherlands, birth cohorts 2008 to 2020
Source: Euro Surveill. 2025 Oct 2;30(39):2500251. doi: 10.2807/1560-7917.ES.2025.30.39.2500251 (PMC12495380; doi:10.2807/1560-7917.ES.2025.30.39.2500251)
Supplement: Supplement [file 25-00251_PIJPERS_Supplement.pdf]

Supplementary files

This supplementary material is hosted by Eurosurveillance as supporting information alongside the article 'The decrease in childhood vaccination coverage in the Netherlands from birth cohort 2008 to 2020 and its sociodemographic determinants', on behalf of the authors, who remain responsible for the accuracy and appropriateness of the content. The same standards for ethics, copyright, attributions and permissions as for the article apply. Supplements are not edited by Eurosurveillance and the journal is not responsible for the maintenance of any links or email addresses provided therein.

Supplementary Table S1. MMR and DTaP-IPV vaccination schedule in the Netherlands in 2022

	Age	Vaccine	Vaccination status	Schedule changes
Phase 1 – Infants	2 months	DTaP-IPV	Primary series	Since the introduction of maternal pertussis vaccination in 2019, this vaccination can be skipped in case the mother is vaccinated.
	3 months	DTaP-IPV	Primary series	-
	5 months	DTaP-IPV	Primary series	-
	11 months	DTaP-IPV booster	Basic immunity	-
	14 months	MMR	Basic immunity	-
Phase 2 – Pre-school	4 years	DTaP-IPV booster	Revaccinated	-
Phase 3 – School age	9 years	DT-IPV	Fully vaccinated	-
	9 years	MMR	Fully vaccinated	-

Abbreviations: MMR *Measles-Mumps-Rubella,* DTaP-IPV *Diphtheria-Tetanus-Pertussis-Poliomyelitis*
Source: National Immunisation Programme | RIVM

Supplementary Table S2. Distribution sociodemographic variables and MMR and DTaP-IPV vaccination coverage per birth cohort

Category	MMR population	Vaccinated		DTaP-IPV population	Vaccinated		Category	MMR population	Vaccinated		DTaP-IPV population	Vaccinated	
	N	N	%	N	N	%		N	N	%	N	N	%
Education level mother high							Country of origin: The Netherlands						
Cohort 2008	55314	53742	97	55265	53619	97	Cohort 2008	118975	114700	96	118968	114613	96
Cohort 2009	58417	56784	97	58371	56674	97	Cohort 2009	118260	114019	96	118263	113889	96
Cohort 2010	60797	59028	97	60762	58905	97	Cohort 2010	116353	112059	96	116365	111827	96
Cohort 2011	67581	65501	97	67542	65440	97	Cohort 2011	112850	108601	96	112844	108497	96
Cohort 2012	67717	65440	97	67658	65372	97	Cohort 2012	108811	104285	96	108804	104218	96
Cohort 2013	67827	65115	96	67780	65335	96	Cohort 2013	105450	100399	95	105445	100864	96
Cohort 2014	70118	66677	95	70060	67062	96	Cohort 2014	106602	100614	94	106617	101408	95
Cohort 2015	69658	65574	94	69604	66144	95	Cohort 2015	102457	95762	93	102444	96678	94
Cohort 2016	71439	67530	95	71381	67673	95	Cohort 2016	102144	95820	94	102136	96169	94
Cohort 2017	71475	68213	95	71430	68078	95	Cohort 2017	100069	94617	95	100063	94538	94
Cohort 2018	72076	68993	96	72031	68821	96	Cohort 2018	97802	92814	95	97783	92759	95
Cohort 2019	73178	69616	95	73148	69522	95	Cohort 2019	97499	92343	95	97475	92208	95
Cohort 2020	73790	68789	93	73753	69406	94	Cohort 2020	97023	89821	93	96990	90830	94
Education level mother intermediate							Country of origin: Europe (excl. NL)						
Cohort 2008	40155	38877	97	40120	38698	96	Cohort 2008	20717	18316	88	21108	17658	84
Cohort 2009	43837	42431	97	43810	42223	96	Cohort 2009	21197	18990	90	21613	18304	85
Cohort 2010	47775	46229	97	47745	46018	96	Cohort 2010	21609	19320	89	21978	18815	86
Cohort 2011	51776	50083	97	51752	49919	96	Cohort 2011	20861	18709	90	21243	18206	86
Cohort 2012	53284	51260	96	53251	51121	96	Cohort 2012	20764	18593	90	21172	18151	86
Cohort 2013	54219	51701	95	54197	51920	96	Cohort 2013	20453	18306	90	20827	17994	86
Cohort 2014	58034	54628	94	58004	55115	95	Cohort 2014	21000	18696	89	21422	18495	86
Cohort 2015	58154	53904	93	58121	54522	94	Cohort 2015	20800	18378	88	21171	18306	86
Cohort 2016	60073	55373	92	60042	55760	93	Cohort 2016	21007	18574	88	21432	18570	87

Cohort 2017	60144	55808	93	60118	55836	93	Cohort 2017	20897	18703	90	21245	18540	87
Cohort 2018	59755	55305	93	59730	55458	93	Cohort 2018	20863	18915	91	21211	18764	88
Cohort 2019	60875	55291	91	60863	55713	92	Cohort 2019	20862	18804	90	21203	18859	89
Cohort 2020	60872	52946	87	60851	54039	89	Cohort 2020	20832	18267	88	21108	18531	88
Education level mother low							Country of origin: Indonesia						
Cohort 2008	19495	18777	96	19472	18591	95	Cohort 2008	7741	7483	97	7738	7466	96
Cohort 2009	20538	19802	96	20521	19545	95	Cohort 2009	7664	7420	97	7665	7424	97
Cohort 2010	20951	20266	97	20921	20042	96	Cohort 2010	7316	7075	97	7321	7086	97
Cohort 2011	23765	23033	97	23742	22866	96	Cohort 2011	6953	6703	96	6952	6689	96
Cohort 2012	23175	22427	97	23166	22281	96	Cohort 2012	6601	6356	96	6610	6364	96
Cohort 2013	21683	20822	96	21647	20815	96	Cohort 2013	6248	5976	96	6250	6002	96
Cohort 2014	21883	20686	95	21812	20838	96	Cohort 2014	6094	5767	95	6100	5825	95
Cohort 2015	20424	18911	93	20374	19123	94	Cohort 2015	5842	5466	94	5849	5518	94
Cohort 2016	19764	18302	93	19747	18456	93	Cohort 2016	5420	5120	94	5423	5148	95
Cohort 2017	17925	16641	93	17898	16648	93	Cohort 2017	5170	4927	95	5178	4938	95
Cohort 2018	17291	15999	93	17279	16040	93	Cohort 2018	4905	4693	96	4907	4690	96
Cohort 2019	15621	14069	90	15612	14239	91	Cohort 2019	4608	4349	94	4617	4389	95
Cohort 2020	14364	12173	85	14354	12500	87	Cohort 2020	4260	3927	92	4267	3971	93
Education level mother unknown							Country of origin: Morocco						
Cohort 2008	75891	69672	92	76539	68438	89	Cohort 2008	8591	8366	97	8587	8279	96
Cohort 2009	67652	62153	92	68390	61023	89	Cohort 2009	8519	8274	97	8518	8199	96
Cohort 2010	60169	55002	91	60865	54063	89	Cohort 2010	8487	8278	98	8484	8216	97
Cohort 2011	41636	37316	90	42393	36575	86	Cohort 2011	8438	8216	97	8451	8190	97
Cohort 2012	36309	32047	88	37116	31496	85	Cohort 2012	8366	8095	97	8380	8091	97
Cohort 2013	31681	27854	88	32409	27446	85	Cohort 2013	8142	7749	95	8147	7802	96
Cohort 2014	28867	25043	87	29633	24780	84	Cohort 2014	8330	7658	92	8342	7815	94
Cohort 2015	25951	22096	85	26586	21947	83	Cohort 2015	8201	7160	87	8197	7324	89
Cohort 2016	24170	20749	86	24851	20624	83	Cohort 2016	8255	7015	85	8261	7133	86
Cohort 2017	22840	19960	87	23482	19801	84	Cohort 2017	8014	6826	85	8015	6858	86

Cohort 2018	21636	19147	88	22196	19036	86	Cohort 2018	7830	6460	83	7830	6534	83
Cohort 2019	21345	18841	88	21912	18891	86	Cohort 2019	8074	5990	74	8071	6237	77
Cohort 2020	20472	17603	86	20893	17905	86	Cohort 2020	8007	5239	65	8006	5480	68
Standardised disposable income household: first quartile							Country of origin: Turkey						
Cohort 2008	29025	27353	94	28978	27030	93	Cohort 2008	6806	6589	97	6821	6504	95
Cohort 2009	28916	27197	94	28844	26831	93	Cohort 2009	6552	6363	97	6559	6302	96
Cohort 2010	28991	27382	94	28925	27075	94	Cohort 2010	6789	6640	98	6793	6571	97
Cohort 2011	27754	26167	94	27697	26002	94	Cohort 2011	6668	6533	98	6682	6502	97
Cohort 2012	26877	25271	94	26838	25112	94	Cohort 2012	6750	6596	98	6765	6584	97
Cohort 2013	25035	23304	93	24969	23263	93	Cohort 2013	6501	6317	97	6505	6307	97
Cohort 2014	24806	22720	92	24730	22820	92	Cohort 2014	6994	6688	96	6997	6713	96
Cohort 2015	22868	20455	89	22782	20626	91	Cohort 2015	6803	6330	93	6784	6402	94
Cohort 2016	22806	20298	89	22735	20382	90	Cohort 2016	7112	6504	91	7097	6556	92
Cohort 2017	21171	18963	90	21096	18817	89	Cohort 2017	6895	6288	91	6883	6308	92
Cohort 2018	20718	18549	90	20673	18540	90	Cohort 2018	6839	6181	90	6828	6233	91
Cohort 2019	19229	16641	87	19223	16834	88	Cohort 2019	6812	5780	85	6813	5955	87
Cohort 2020	18502	15082	82	18442	15414	84	Cohort 2020	6527	5174	79	6512	5363	82
Standardised disposable income household: second quartile							Country of origin: Suriname						
Cohort 2008	58480	56286	96	58438	55989	96	Cohort 2008	6074	5915	97	6077	5812	96
Cohort 2009	57046	54864	96	57025	54642	96	Cohort 2009	6187	6015	97	6188	5933	96
Cohort 2010	54402	52197	96	54365	51910	95	Cohort 2010	6236	6080	97	6237	6018	96
Cohort 2011	51616	49462	96	51598	49257	95	Cohort 2011	6029	5869	97	6038	5846	97
Cohort 2012	49035	46768	95	49003	46585	95	Cohort 2012	5846	5680	97	5857	5653	97
Cohort 2013	46153	43581	94	46137	43705	95	Cohort 2013	5758	5545	96	5760	5552	96

Cohort 2014	47911	44643	93	47889	45000	94	Cohort 2014	5678	5436	96	5681	5452	96
Cohort 2015	47018	43119	92	46980	43578	93	Cohort 2015	5708	5338	94	5711	5408	95
Cohort 2016	48674	44501	91	48645	44747	92	Cohort 2016	5721	5281	92	5730	5321	93
Cohort 2017	47119	43384	92	47074	43362	92	Cohort 2017	5557	5213	94	5564	5227	94
Cohort 2018	46632	42836	92	46594	42773	92	Cohort 2018	5468	5070	93	5472	5088	93
Cohort 2019	46870	42352	90	46879	42575	91	Cohort 2019	5485	4955	90	5492	5022	91
Cohort 2020	44864	38765	86	44827	39585	88	Cohort 2020	5490	4705	86	5498	4830	88
Standardised disposable income household: third quartile							Country of origin: The Dutch Caribbean						
Cohort 2008	56445	54740	97	56385	54556	97	Cohort 2008	3597	3362	93	3604	3325	92
Cohort 2009	57012	55494	97	56981	55253	97	Cohort 2009	3498	3326	95	3508	3301	94
Cohort 2010	57237	55666	97	57196	55467	97	Cohort 2010	3591	3390	94	3596	3370	94
Cohort 2011	57771	56244	97	57755	56122	97	Cohort 2011	3457	3288	95	3482	3294	95
Cohort 2012	57084	55284	97	57062	55153	97	Cohort 2012	3460	3265	94	3483	3266	94
Cohort 2013	56781	54708	96	56762	54872	97	Cohort 2013	3439	3246	94	3459	3282	95
Cohort 2014	58602	55909	95	58520	56206	96	Cohort 2014	3642	3405	93	3652	3449	94
Cohort 2015	58006	54571	94	57902	55017	95	Cohort 2015	3527	3248	92	3526	3296	93
Cohort 2016	58542	55297	94	58464	55447	95	Cohort 2016	3667	3326	91	3681	3365	91
Cohort 2017	59987	57072	95	59938	56987	95	Cohort 2017	3505	3230	92	3514	3237	92
Cohort 2018	59755	57031	95	59691	57015	96	Cohort 2018	3629	3300	91	3634	3313	91
Cohort 2019	60585	57331	95	60553	57396	95	Cohort 2019	3576	3164	88	3585	3207	89
Cohort 2020	60592	55798	92	60547	56468	93	Cohort 2020	3738	3125	84	3733	3224	86
Standardised disposable income household: fourth quartile							Country of origin: Other, Africa						
Cohort 2008	39762	38652	97	39717	38368	97	Cohort 2008	5522	4981	90	5532	4897	89
Cohort 2009	40547	39409	97	40504	39149	97	Cohort 2009	5615	5169	92	5630	5060	90

Cohort 2010	42067	41001	97	42027	40773	97	Cohort 2010	5860	5490	94	5881	5426	92
Cohort 2011	41049	39943	97	41008	39755	97	Cohort 2011	5864	5564	95	5887	5554	94
Cohort 2012	41087	39862	97	41026	39689	97	Cohort 2012	5941	5608	94	5964	5605	94
Cohort 2013	41634	40244	97	41556	40214	97	Cohort 2013	5794	5499	95	5821	5480	94
Cohort 2014	42141	40325	96	42071	40450	96	Cohort 2014	5954	5488	92	5953	5552	93
Cohort 2015	41288	39221	95	41197	39410	96	Cohort 2015	5789	5304	92	5797	5377	93
Cohort 2016	41122	39226	95	41056	39220	96	Cohort 2016	6031	5573	92	6060	5626	93
Cohort 2017	40424	38902	96	40373	38764	96	Cohort 2017	6163	5748	93	6148	5754	94
Cohort 2018	40354	38880	96	40254	38722	96	Cohort 2018	6721	6297	94	6711	6301	94
Cohort 2019	41085	39390	96	41029	39255	96	Cohort 2019	7015	6409	91	7023	6462	92
Cohort 2020	43395	40645	94	43314	40942	95	Cohort 2020	6784	5908	87	6774	6076	90
Standardised disposable income household: unknown							Country of origin: Other, America/Oceania						
Cohort 2008	7143	4037	57	7878	3403	43	Cohort 2008	4524	4007	89	4559	3739	82
Cohort 2009	6923	4206	61	7738	3590	46	Cohort 2009	4566	4086	89	4613	3827	83
Cohort 2010	6995	4279	61	7780	3803	49	Cohort 2010	4636	4179	90	4670	3939	84
Cohort 2011	6568	4117	63	7371	3664	50	Cohort 2011	4724	4308	91	4782	4100	86
Cohort 2012	6402	3989	62	7262	3731	51	Cohort 2012	4642	4217	91	4703	4006	85
Cohort 2013	5807	3655	63	6609	3462	52	Cohort 2013	4641	4215	91	4704	4079	87
Cohort 2014	5442	3437	63	6299	3319	53	Cohort 2014	5002	4550	91	5037	4433	88
Cohort 2015	5007	3119	62	5824	3105	53	Cohort 2015	4944	4460	90	5002	4402	88
Cohort 2016	4302	2632	61	5121	2717	53	Cohort 2016	4892	4475	91	4929	4421	90
Cohort 2017	3683	2301	62	4447	2433	55	Cohort 2017	4988	4626	93	5036	4549	90
Cohort 2018	3299	2148	65	4024	2305	57	Cohort 2018	4977	4652	93	5012	4620	92
Cohort 2019	3250	2103	65	3851	2305	60	Cohort 2019	5195	4816	93	5220	4800	92
Cohort 2020	2145	1221	57	2721	1441	53	Cohort 2020	5147	4640	90	5164	4683	91
Income source mother: Job							Country of origin: Other, Asia						

in employment													
Cohort 2008	126454	123593	98	126369	123045	97	Cohort 2008	8308	7349	88	8330	7046	85
Cohort 2009	126154	123321	98	126070	122821	97	Cohort 2009	8386	7508	90	8448	7215	85
Cohort 2010	123131	120277	98	123051	119800	97	Cohort 2010	8815	8014	91	8888	7753	87
Cohort 2011	118906	116056	98	118860	115783	97	Cohort 2011	8914	8142	91	9005	7909	88
Cohort 2012	115778	112628	97	115696	112363	97	Cohort 2012	9304	8479	91	9374	8319	89
Cohort 2013	113800	110078	97	113734	110342	97	Cohort 2013	8984	8240	92	9039	8141	90
Cohort 2014	118000	113223	96	117904	113830	97	Cohort 2014	9606	8732	91	9635	8645	90
Cohort 2015	116902	110997	95	116815	111859	96	Cohort 2015	10116	9039	89	10106	9008	89
Cohort 2016	119594	113604	95	119476	113932	95	Cohort 2016	11197	10266	92	11191	10191	91
Cohort 2017	117271	112132	96	117171	112008	96	Cohort 2017	11126	10444	94	11196	10403	93
Cohort 2018	116973	112118	96	116832	111932	96	Cohort 2018	11724	11062	94	11763	11044	94
Cohort 2019	119930	114012	95	119855	114016	95	Cohort 2019	11893	11207	94	11943	11213	94
Cohort 2020	121259	112354	93	121142	113663	94	Cohort 2020	11690	10705	92	11716	10842	93
Income source mother: self-employed							Level of urbanisation: Not urbanised						
Cohort 2008	13017	12196	94	12997	12202	94	Cohort 2008	30407	28842	95	30380	28741	95
Cohort 2009	13026	12134	93	13014	12126	93	Cohort 2009	29830	28262	95	29809	28184	95
Cohort 2010	13209	12289	93	13198	12289	93	Cohort 2010	28743	27173	95	28717	27038	94
Cohort 2011	13170	12257	93	13161	12250	93	Cohort 2011	27401	25862	94	27385	25765	94
Cohort 2012	13485	12462	92	13470	12434	92	Cohort 2012	26170	24584	94	26157	24505	94
Cohort 2013	13553	12389	91	13541	12430	92	Cohort 2013	24684	23067	93	24672	23144	94
Cohort 2014	14195	12769	90	14180	12880	91	Cohort 2014	25185	23260	92	25166	23437	93
Cohort 2015	14146	12555	89	14125	12695	90	Cohort 2015	25053	22900	91	25028	23138	92
Cohort 2016	14234	12583	88	14227	12642	89	Cohort 2016	25256	23109	91	25235	23178	92
Cohort 2017	13898	12553	90	13895	12515	90	Cohort 2017	25079	23189	92	25070	23161	92
Cohort 2018	14209	12776	90	14198	12742	90	Cohort 2018	25503	23526	92	25487	23498	92
Cohort 2019	14451	12807	89	14447	12835	89	Cohort 2019	25696	23661	92	25691	23615	92
Cohort 2020	13657	11567	85	13638	11739	86	Cohort 2020	25935	23283	90	26043	23626	91

Income source mother: Recipient benefit							Level of urbanisation: hardly						
Cohort 2008	19690	18856	96	19658	18638	95	Cohort 2008	35156	33954	97	35124	33772	96
Cohort 2009	20548	19798	96	20529	19555	95	Cohort 2009	33737	32645	97	33709	32541	97
Cohort 2010	22681	22025	97	22645	21775	96	Cohort 2010	32816	31682	97	32784	31570	96
Cohort 2011	23139	22405	97	23109	22209	96	Cohort 2011	32141	31009	96	32122	30927	96
Cohort 2012	22606	21828	97	22596	21716	96	Cohort 2012	31172	29899	96	31155	29847	96
Cohort 2013	21541	20588	96	21476	20571	96	Cohort 2013	28701	27229	95	28675	27270	95
Cohort 2014	21549	20074	93	21428	20226	94	Cohort 2014	30067	28274	94	30039	28437	95
Cohort 2015	20182	18221	90	20012	18418	92	Cohort 2015	29083	26919	93	29040	27195	94
Cohort 2016	20391	18445	90	20277	18564	92	Cohort 2016	29354	27287	93	29316	27382	93
Cohort 2017	21840	19957	91	21768	19884	91	Cohort 2017	29048	27290	94	29010	27237	94
Cohort 2018	21259	19380	91	21196	19433	92	Cohort 2018	28487	26938	95	28462	26900	95
Cohort 2019	19270	16893	88	19245	17139	89	Cohort 2019	28388	26730	94	28374	26720	94
Cohort 2020	18512	15132	82	18462	15580	84	Cohort 2020	28431	26032	92	28475	26384	93
Income source mother: Recipient benefit							Level of urbanisation: moderately						
Cohort 2008	1415	1334	94	1413	1334	94	Cohort 2008	37879	36662	97	37859	36512	96
Cohort 2009	1590	1505	95	1586	1511	95	Cohort 2009	38302	37100	97	38265	36884	96
Cohort 2010	1370	1304	95	1369	1302	95	Cohort 2010	37074	35978	97	37041	35752	97
Cohort 2011	1038	985	95	1037	978	94	Cohort 2011	35596	34490	97	35577	34401	97
Cohort 2012	780	738	95	779	730	94	Cohort 2012	34224	33076	97	34180	32933	96
Cohort 2013	897	827	92	897	841	94	Cohort 2013	32598	31297	96	32554	31358	96
Cohort 2014	846	777	92	844	782	93	Cohort 2014	33089	31443	95	33038	31643	96
Cohort 2015	604	536	89	601	544	91	Cohort 2015	32237	30252	94	32174	30429	95
Cohort 2016	533	481	90	531	478	90	Cohort 2016	32520	30608	94	32477	30698	95
Cohort 2017	473	428	90	470	425	90	Cohort 2017	31883	30169	95	31848	30099	95

Cohort 2018	456	417	91	456	416	91	Cohort 2018	31504	29796	95	31444	29763	95
Cohort 2019	306	277	91	306	278	91	Cohort 2019	31718	29740	94	31690	29728	94
Cohort 2020	267	218	82	267	222	83	Cohort 2020	31940	29132	91	31960	29559	92
Income source mother: Student							Level of urbanisation: strongly						
Cohort 2008	3125	2990	96	3116	2964	95	Cohort 2008	45514	44160	97	45452	43932	97
Cohort 2009	3337	3200	96	3328	3146	95	Cohort 2009	45542	44147	97	45486	43851	96
Cohort 2010	3222	3109	96	3211	3072	96	Cohort 2010	46811	45433	97	46754	45143	97
Cohort 2011	3228	3119	97	3220	3091	96	Cohort 2011	44261	42967	97	44212	42779	97
Cohort 2012	3046	2926	96	3040	2895	95	Cohort 2012	44469	43021	97	44429	42861	96
Cohort 2013	2808	2690	96	2802	2701	96	Cohort 2013	43881	42094	96	43826	42195	96
Cohort 2014	2619	2446	93	2610	2471	95	Cohort 2014	45047	42750	95	44962	42956	96
Cohort 2015	2352	2111	90	2342	2147	92	Cohort 2015	44263	41417	94	44158	41721	94
Cohort 2016	2328	2087	90	2320	2110	91	Cohort 2016	44851	41931	93	44747	42040	94
Cohort 2017	2361	2177	92	2353	2185	93	Cohort 2017	44638	42143	94	44557	42042	94
Cohort 2018	2365	2182	92	2358	2175	92	Cohort 2018	43655	41331	95	43560	41244	95
Cohort 2019	2187	1958	90	2185	1980	91	Cohort 2019	43668	40646	93	43620	40773	93
Cohort 2020	2452	2058	84	2437	2118	87	Cohort 2020	42402	38288	90	42430	38855	92
Income source mother: Other							Level of urbanisation: Extremely						
Cohort 2008	19919	18048	91	19857	17742	89	Cohort 2008	35217	33860	96	35137	33365	95
Cohort 2009	18845	17077	91	18794	16771	89	Cohort 2009	36689	35369	96	36632	34899	95
Cohort 2010	19074	17316	91	19017	17030	90	Cohort 2010	37904	36619	97	37836	36279	96
Cohort 2011	18545	16928	91	18489	16738	91	Cohort 2011	39261	37956	97	39192	37661	96
Cohort 2012	18261	16592	91	18197	16344	90	Cohort 2012	38490	37083	96	38412	36789	96
Cohort 2013	16905	15217	90	16848	15120	90	Cohort 2013	40222	38618	96	40142	38505	96
Cohort 2014	16239	14331	88	16199	14307	88	Cohort 2014	40615	38378	94	40506	38470	95
Cohort 2015	15036	13044	87	14972	13024	87	Cohort 2015	39041	36325	93	38904	36535	94
Cohort 2016	14089	12174	86	14052	12085	86	Cohort 2016	39588	36742	93	39504	36792	93

Cohort 2017	12925	11156	86	12864	10981	85	Cohort 2017	38442	35864	93	38333	35687	93
Cohort 2018	12311	10540	86	12239	10445	85	Cohort 2018	38773	36102	93	38662	35978	93
Cohort 2019	11882	9963	84	11861	9975	84	Cohort 2019	38869	35368	91	38843	35617	92
Cohort 2020	11745	9322	79	11669	9460	81	Cohort 2020	38102	33116	87	38062	33624	88
Income source mother: unknown							Level of urbanisation: Unknown						
Cohort 2008	7235	4051	56	7986	3421	43	Cohort 2008	6682	3590	54	7444	3024	41
Cohort 2009	6944	4135	60	7771	3535	45	Cohort 2009	6344	3647	57	7191	3106	43
Cohort 2010	7005	4205	60	7802	3760	48	Cohort 2010	6344	3640	57	7161	3246	45
Cohort 2011	6732	4183	62	7553	3751	50	Cohort 2011	6098	3649	60	6941	3267	47
Cohort 2012	6529	4000	61	7413	3788	51	Cohort 2012	5960	3511	59	6858	3335	49
Cohort 2013	5906	3703	63	6735	3511	52	Cohort 2013	5324	3187	60	6164	3044	49
Cohort 2014	5454	3414	63	6344	3299	52	Cohort 2014	4899	2929	60	5798	2852	49
Cohort 2015	4965	3021	61	5818	3049	52	Cohort 2015	4510	2672	59	5381	2718	51
Cohort 2016	4277	2580	60	5138	2702	53	Cohort 2016	3877	2277	59	4742	2423	51
Cohort 2017	3616	2219	61	4407	2365	54	Cohort 2017	3294	1967	60	4110	2137	52
Cohort 2018	3185	2031	64	3957	2212	56	Cohort 2018	2836	1751	62	3621	1972	54
Cohort 2019	2993	1907	64	3636	2142	59	Cohort 2019	2680	1672	62	3317	1912	58
Cohort 2020	1606	860	54	2236	1068	48	Cohort 2020	2688	1660	62	2881	1802	63
Family size: 1-3 children							Generation: Dutch origin						
Cohort 2008	172545	167376	97	172331	166275	96	Cohort 2008	118975	114700	96	118970	114615	96
Cohort 2009	172826	167650	97	172634	166559	96	Cohort 2009	118260	114019	96	118265	113891	96
Cohort 2010	172303	167205	97	172089	166132	97	Cohort 2010	116353	112059	96	116368	111830	96
Cohort 2011	167861	162776	97	167683	162026	97	Cohort 2011	112850	108601	96	112850	108502	96
Cohort 2012	164117	158579	97	163922	157936	96	Cohort 2012	108811	104285	96	108810	104224	96
Cohort 2013	159857	153400	96	159633	153537	96	Cohort 2013	105450	100399	95	105451	100870	96
Cohort 2014	163176	154938	95	162898	155677	96	Cohort 2014	106602	100614	94	106619	101410	95
Cohort 2015	159037	148994	94	158693	150077	95	Cohort 2015	102457	95762	93	102447	96681	94
Cohort 2016	160491	150498	94	160217	150847	94	Cohort 2016	102144	95820	94	102140	96173	94
Cohort 2017	158065	149396	95	157800	148990	94	Cohort 2017	100069	94617	95	100064	94539	94

Cohort 2018	156588	148292	95	156281	147957	95	Cohort 2018	97802	92814	95	97785	92759	95
Cohort 2019	157085	147094	94	156945	147294	94	Cohort 2019	97499	92343	95	97476	92209	95
Cohort 2020	155815	141583	91	155870	143489	92	Cohort 2020	97023	89821	93	96991	90831	94
Family size: 4 or more children							Generation: Migrant or child of first generation migrant(s)						
Cohort 2008	11358	9893	87	11350	9853	87	Cohort 2008	51981	47029	90	52448	45470	87
Cohort 2009	11032	9655	88	11026	9594	87	Cohort 2009	51511	47036	91	52052	45526	87
Cohort 2010	10722	9397	88	10713	9359	87	Cohort 2010	52194	47890	92	52684	46676	89
Cohort 2011	10492	9249	88	10491	9246	88	Cohort 2011	51078	47107	92	51667	46090	89
Cohort 2012	10109	8820	87	10106	8744	87	Cohort 2012	50467	46355	92	51082	45506	89
Cohort 2013	9949	8662	87	9941	8680	87	Cohort 2013	48420	44413	92	48963	43835	90
Cohort 2014	10496	8902	85	10469	8992	86	Cohort 2014	49846	45238	91	50338	44933	89
Cohort 2015	10356	8599	83	10318	8706	84	Cohort 2015	48906	43659	89	49296	43634	89
Cohort 2016	10776	8960	83	10739	9003	84	Cohort 2016	49557	44413	90	50030	44389	89
Cohort 2017	10708	9025	84	10683	8982	84	Cohort 2017	48639	44122	91	49069	43869	89
Cohort 2018	10975	9128	83	10961	9147	83	Cohort 2018	48802	44463	91	49168	44279	90
Cohort 2019	10880	8810	81	10872	8896	82	Cohort 2019	48973	43782	89	49387	44108	89
Cohort 2020	10736	8108	76	10743	8315	77	Cohort 2020	47729	40998	86	47969	41723	87
Family size: Institutional							Generation: Child of second generation migrant(s)						
Cohort 2008	265	202	76	261	182	70	Cohort 2008	19899	19339	97	19908	19256	97
Cohort 2009	241	218	90	238	204	86	Cohort 2009	20673	20115	97	20690	20039	97
Cohort 2010	330	288	87	330	288	87	Cohort 2010	21145	20576	97	21164	20518	97
Cohort 2011	317	269	85	317	264	83	Cohort 2011	20830	20225	97	20855	20200	97
Cohort 2012	312	275	88	311	261	84	Cohort 2012	21207	20534	97	21226	20533	97
Cohort 2013	292	255	87	297	257	87	Cohort 2013	21540	20680	96	21549	20804	97
Cohort 2014	347	280	81	347	277	80	Cohort 2014	22454	21182	94	22482	21446	95
Cohort 2015	298	231	78	295	236	80	Cohort 2015	22824	21064	92	22847	21407	94

Cohort 2016	334	250	75	340	256	75	Cohort 2016	23745	21721	91	23774	21942	92
Cohort 2017	337	253	75	342	260	76	Cohort 2017	23676	21883	92	23710	21945	93
Cohort 2018	383	291	76	383	286	75	Cohort 2018	24154	22167	92	24200	22308	92
Cohort 2019	425	287	68	424	284	67	Cohort 2019	24547	21692	88	24580	22036	90
Cohort 2020	378	263	70	376	257	68	Cohort 2020	24746	20692	84	24809	21277	86
Family size: unknown							Day care attendance: No						
Cohort 2008	6687	3597	54	7454	3036	41	Cohort 2008	88086	80259	91	88723	79035	89
Cohort 2009	6345	3647	57	7194	3108	43	Cohort 2009	85088	77790	91	85826	76598	89
Cohort 2010	6337	3635	57	7161	3249	45	Cohort 2010	86964	79657	92	87645	78557	90
Cohort 2011	6088	3639	60	6938	3264	47	Cohort 2011	90229	83143	92	90960	82231	90
Cohort 2012	5947	3500	59	6852	3329	49	Cohort 2012	88910	81565	92	89693	80855	90
Cohort 2013	5312	3175	60	6162	3042	49	Cohort 2013	84040	76559	91	84742	76419	90
Cohort 2014	4883	2914	60	5795	2849	49	Cohort 2014	77555	69284	89	78325	69565	89
Cohort 2015	4496	2661	59	5379	2717	51	Cohort 2015	68283	59536	87	68965	60024	87
Cohort 2016	3845	2246	58	4725	2407	51	Cohort 2016	60695	52341	86	61433	52632	86
Cohort 2017	3274	1948	59	4103	2131	52	Cohort 2017	54559	47194	87	55237	47104	85
Cohort 2018	2812	1733	62	3611	1965	54	Cohort 2018	53172	45831	86	53798	46007	86
Cohort 2019	2629	1626	62	3294	1891	57	Cohort 2019	50469	42142	84	51076	42741	84
Cohort 2020	2569	1557	61	2862	1789	63	Cohort 2020	45981	35993	78	46490	37171	80
Day care attendance: yes													
Cohort 2008	102713	100753	98	102617	100258	98							
Cohort 2009	105331	103355	98	105241	102842	98							
Cohort 2010	102713	100853	98	102632	100456	98							
Cohort 2011	94513	92776	98	94453	92555	98							
Cohort 2012	91568	89602	98	91491	89408	98							
Cohort 2013	91365	88928	97	91286	89092	98							
Cohort 2014	101346	97749	96	101183	98229	97							
Cohort 2015	105899	100944	95	105715	101707	96							
Cohort 2016	114749	109611	96	114586	109879	96							
Cohort 2017	117822	113425	96	117688	113256	96							

Cohort 2018	117585	113612	97	117437	113348	97
Cohort 2019	120547	115672	96	120456	115621	96
Cohort 2020	123517	115518	94	123353	116671	95

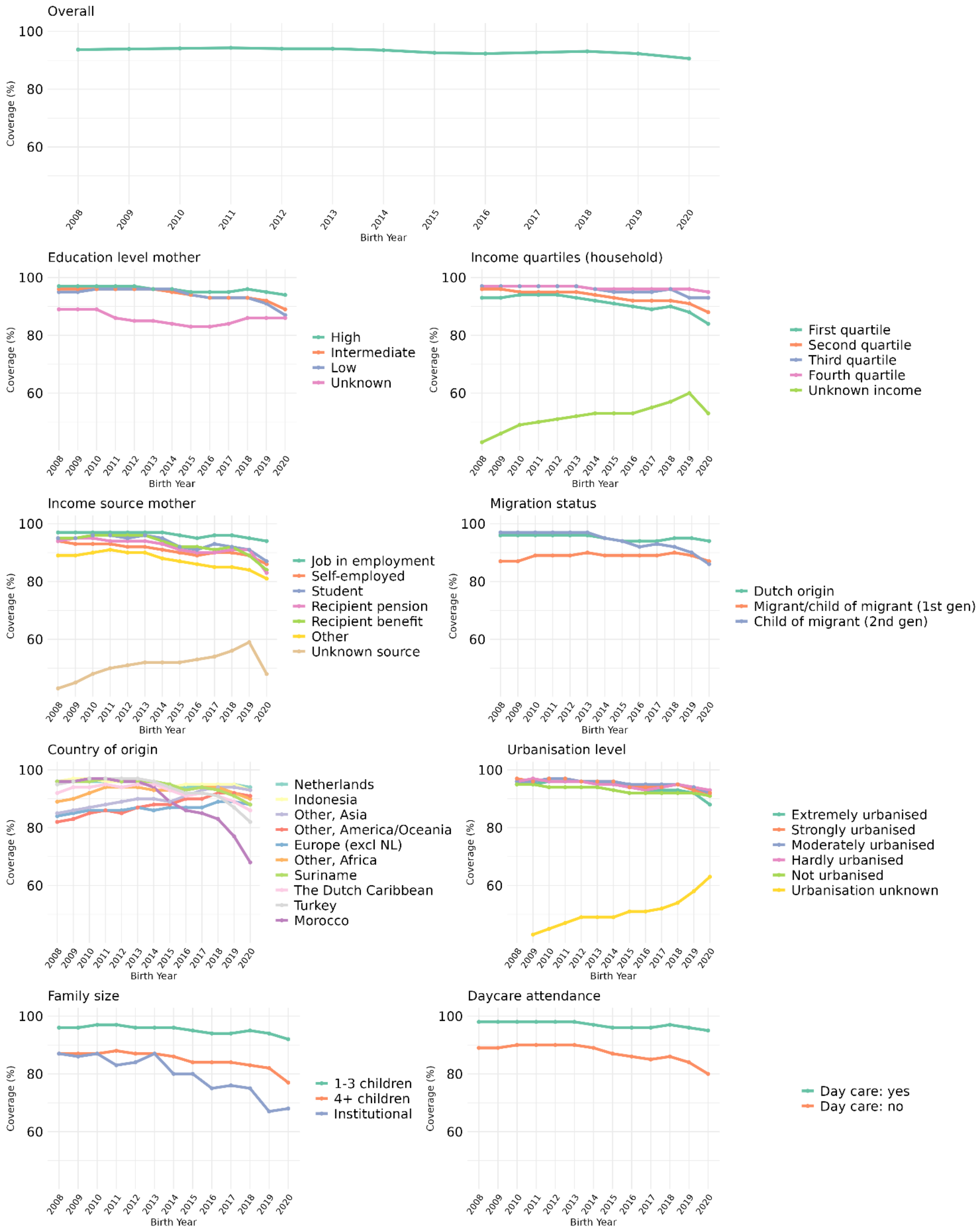

Supplementary Figure S3. Crude DTaP-IPV vaccination coverage by sociodemographic determinant

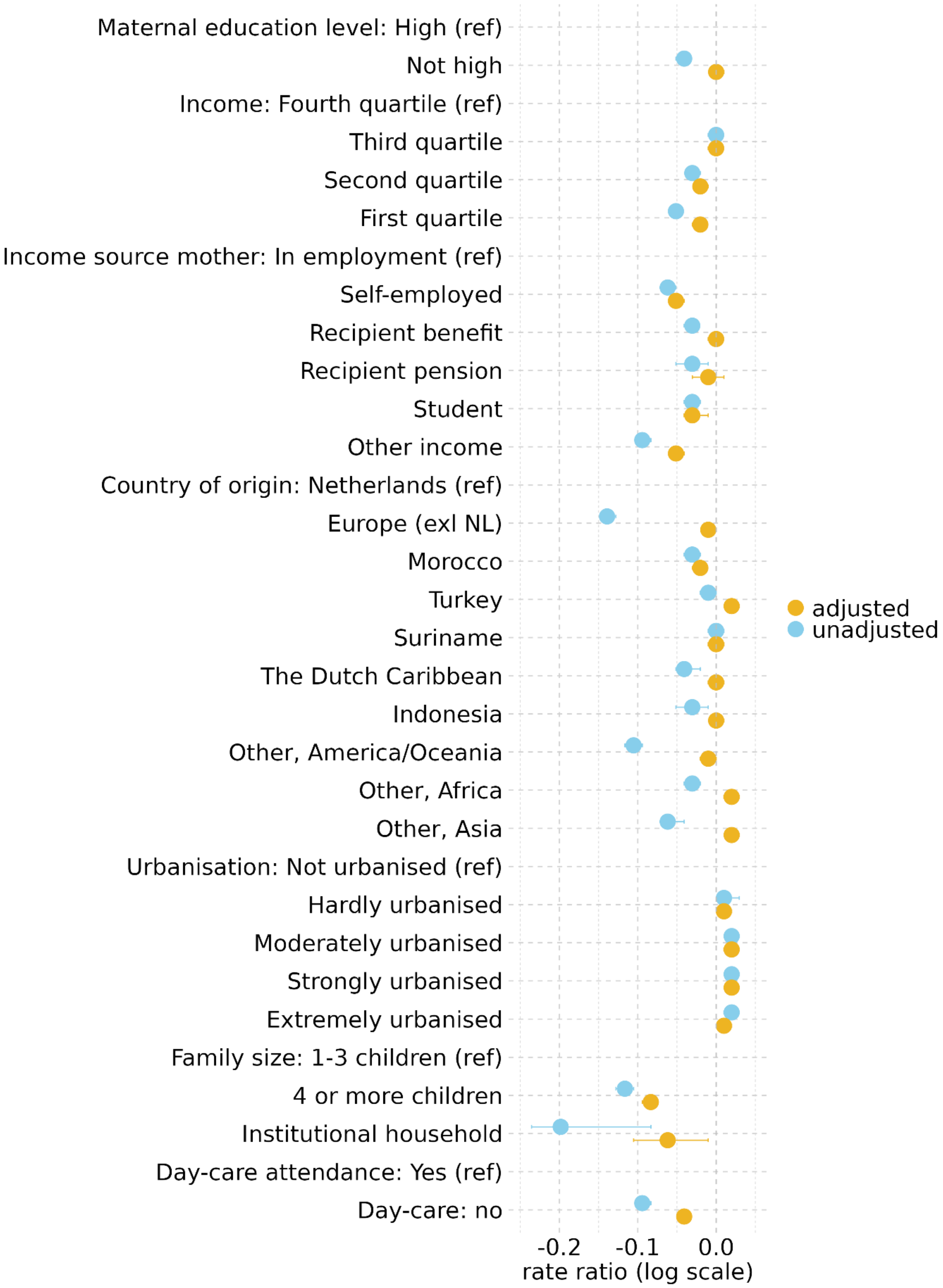

Supplementary Figure S4. DTaP-IPV univariable and multivariable Poisson regression model – unadjusted and adjusted RRs

In addition to all the variables listed, the RRs are also adjusted for birth cohort.

Supplementary Table S5. Multivariable Poisson regression model with interaction terms for birth cohort * sociodemographic variable for both MMR and DTaP-IPV vaccination

	MMR vaccination model	Sensitivity analysis*:		DTaP-IPV vaccination model	Sensitivity analysis*:	
Category	aRR (95% CI)	p-value	aRR (95% CI)	aRR (95% CI)	p-value	aRR (95% CI)
Birth_year2009	1.00 (0.97 - 1.02)	0.807	1.00 (0.97 - 1.02)	1.00 (0.98 - 1.02)	0.980	1.00 (0.98 -1.02)
Birth_year2010	1.00 (0.97 - 1.02)	0.767	1.00 (0.97 - 1.02)	1.00 (0.97 - 1.02)	0.742	1.00 (0.97 -1.02)
Birth_year2011	0.99 (0.97 - 1.02)	0.542	0.99 (0.97 - 1.02)	0.99 (0.97 - 1.02)	0.598	0.99 (0.97 -1.02)
Birth_year2012	0.99 (0.97 - 1.01)	0.374	0.99 (0.97 - 1.01)	0.99 (0.97 - 1.01)	0.483	0.99 (0.97 -1.01)
Birth_year2013	0.99 (0.96 - 1.01)	0.256	0.99 (0.96 - 1.01)	0.99 (0.97 - 1.02)	0.517	0.99 (0.97 -1.01)
Birth_year2014	0.98 (0.96 - 1.00)	0.074	0.98 (0.95 - 1.00)	0.99 (0.97 - 1.01)	0.293	0.99 (0.96 -1.01)
Birth_year2015	0.97 (0.95 - 1.00)	0.027	0.97 (0.95 - 1.00)	0.98 (0.96 - 1.01)	0.186	0.98 (0.96 -1.01)
Birth_year2016	0.98 (0.96 - 1.00)	0.083	0.98 (0.96 - 1.00)	0.98 (0.96 - 1.01)	0.141	0.98 (0.96 -1.00)
Birth_year2017	0.99 (0.96 - 1.01)	0.237	0.99 (0.96 - 1.01)	0.99 (0.96 - 1.01)	0.272	0.98 (0.96 -1.01)
Birth_year2018	0.99 (0.96 - 1.01)	0.228	0.98 (0.96 - 1.01)	0.99 (0.96 - 1.01)	0.219	0.98 (0.96 -1.01)
Birth_year2019	0.99 (0.97 - 1.02)	0.598	0.99 (0.97 - 1.01)	0.99 (0.97 - 1.01)	0.324	0.99 (0.96 -1.01)
Birth_year2020	0.98 (0.96 - 1.00)	0.094	0.98 (0.96 - 1.00)	0.99 (0.96 - 1.01)	0.253	0.98 (0.96 -1.01)
High_education_motherNo	1.00 (0.99 - 1.02)	0.475	1.00 (0.99 - 1.02)	1.00 (0.99 - 1.01)	0.977	1.00 (0.99 -1.01)
SDIhh_quartileFirst quartile	0.99 (0.97 - 1.01)	0.222	0.99 (0.97 - 1.01)	0.99 (0.97 - 1.01)	0.264	0.99 (0.97 -1.01)
SDIhh_quartileSecond quartile	0.99 (0.98 - 1.01)	0.335	0.99 (0.98 - 1.01)	1.00 (0.98 - 1.01)	0.645	1.00 (0.98 -1.01)
SDIhh_quartileThird quartile	1.00 (0.98 - 1.01)	0.525	1.00 (0.98 - 1.01)	1.00 (0.99 - 1.01)	0.996	1.00 (0.99 -1.01)
SDIhh_quartileUnknown	NA	NA	0.87 (0.81 - 0.93)	NA	NA	0.79 (0.72 -0.85)
Country_origin_thirdgenEurope (excl NL)	1.00 (0.98 - 1.01)	0.593	0.99 (0.97 - 1.00)	0.99 (0.97 - 1.00)	0.157	0.97 (0.95 -0.98)
Country_origin_thirdgenIndonesia	1.00 (0.98 - 1.03)	0.694	1.00 (0.98 - 1.03)	1.00 (0.98 - 1.03)	0.830	1.00 (0.98 -1.03)
Country_origin_thirdgenMorocco	1.05 (1.02 - 1.07)	<0.001	1.05 (1.02 - 1.07)	1.04 (1.02 - 1.07)	0.001	1.04 (1.02 -1.07)
Country_origin_thirdgenOther, Africa	1.00 (0.97 - 1.03)	0.787	0.98 (0.95 - 1.01)	0.98 (0.95 - 1.01)	0.226	0.98 (0.95 -1.01)
Country_origin_thirdgenOther, America/Oceania	0.99 (0.96 - 1.03)	0.699	1.01 (0.98 - 1.04)	0.98 (0.95 - 1.02)	0.387	0.97 (0.93 -1.00)
Country_origin_thirdgenOther, Asia	1.01 (0.99 - 1.04)	0.308	1.00 (0.98 - 1.03)	1.00 (0.97 - 1.02)	0.781	0.99 (0.96 -1.01)
Country_origin_thirdgenSuriname	1.01 (0.99 - 1.04)	0.295	1.02 (0.99 - 1.04)	1.00 (0.98 - 1.03)	0.848	1.00 (0.98 -1.03)
Country_origin_thirdgenThe Dutch Caribbean	1.00 (0.97 - 1.04)	0.846	1.01 (0.97 - 1.04)	0.99 (0.96 - 1.03)	0.772	1.01 (0.97 -1.05)
Country_origin_thirdgenTurkey	1.03 (1.01 - 1.06)	0.011	1.03 (1.01 - 1.06)	1.03 (1.00 - 1.05)	0.057	1.03 (1.00 -1.05)
SES.mother5Self-employed	0.97 (0.95 - 0.99)	0.001	0.97 (0.95 - 0.99)	0.97 (0.96 - 0.99)	0.007	0.98 (0.96 -0.99)
SES.mother5Recipient benefit	0.99 (0.97 - 1.01)	0.229	0.99 (0.98 - 1.01)	0.99 (0.97 - 1.01)	0.178	0.99 (0.97 -1.01)
SES.mother5Recipient benefit	0.98 (0.93 - 1.04)	0.504	0.98 (0.93 - 1.04)	0.99 (0.94 - 1.04)	0.677	0.99 (0.94 -1.05)
SES.mother5Child/student	0.98 (0.94 - 1.02)	0.261	0.98 (0.94 - 1.02)	0.98 (0.95 - 1.02)	0.386	0.98 (0.95 -1.02)

SES.mother5Other	0.95 (0.93 - 0.97)	<0.001	0.95 (0.94 - 0.97)	0.95 (0.93 - 0.96)	<0.001	0.95 (0.93 -0.97)
SES.mother5Unknown	NA	NA	0.87 (0.79 - 0.94)	NA	NA	0.81 (0.72 -0.89)
UrbanisationExtremely urbanised	1.01 (0.99 - 1.02)	0.469	1.01 (0.99 - 1.02)	1.00 (0.99 - 1.02)	0.806	1.00 (0.99 -1.02)
UrbanisationHardly urbanised	1.01 (0.99 - 1.03)	0.199	1.01 (0.99 - 1.03)	1.01 (0.99 - 1.03)	0.266	1.01 (0.99 -1.02)
UrbanisationModerately urbanised	1.01 (1.00 - 1.03)	0.151	1.01 (1.00 - 1.03)	1.01 (1.00 - 1.03)	0.125	1.01 (1.00 -1.03)
UrbanisationStrongly urbanised	1.01 (1.00 - 1.03)	0.068	1.01 (1.00 - 1.03)	1.02 (1.00 - 1.03)	0.040	1.02 (1.00 -1.03)
UrbanisationUnknown	NA	NA	0.74 (0.32 - 1.16)	NA	NA	0.61 (0.15 -1.07)
family_size4 or more children	0.91 (0.89 - 0.93)	<0.001	0.91 (0.89 - 0.93)	0.91 (0.90 - 0.93)	<0.001	0.91 (0.89 -0.94)
family_sizeInstitutional	0.90 (0.75 - 1.06)	0.206	0.86 (0.72 - 1.00)	0.86 (0.72 - 1.03)	0.097	0.83 (0.68 -0.97)
Family_sizeUnknown	NA	NA	0.99 (0.58 - 1.41)	NA	NA	1.10 (0.64 -1.55)
Daycare2No daycare	0.98 (0.97 - 0.99)	<0.001	0.97 (0.96 - 0.98)	0.98 (0.97 - 0.99)	<0.001	0.97 (0.96 -0.98)
Birth_year2009:High_education_motherNo	1.00 (0.98 - 1.02)	0.997	1.00 (0.98 - 1.02)	1.00 (0.98 - 1.02)	0.965	1.00 (0.98 -1.02)
Birth_year2010:High_education_motherNo	1.00 (0.99 - 1.02)	0.781	1.00 (0.99 - 1.02)	1.00 (0.99 - 1.02)	0.698	1.00 (0.99 -1.02)
Birth_year2011:High_education_motherNo	1.00 (0.99 - 1.02)	0.660	1.00 (0.99 - 1.02)	1.00 (0.99 - 1.02)	0.630	1.00 (0.99 -1.02)
Birth_year2012:High_education_motherNo	1.00 (0.99 - 1.02)	0.754	1.00 (0.99 - 1.02)	1.00 (0.99 - 1.02)	0.702	1.00 (0.99 -1.02)
Birth_year2013:High_education_motherNo	1.01 (0.99 - 1.02)	0.520	1.01 (0.99 - 1.02)	1.01 (0.99 - 1.02)	0.432	1.01 (0.99 -1.02)
Birth_year2014:High_education_motherNo	1.01 (0.99 - 1.02)	0.404	1.01 (0.99 - 1.02)	1.01 (0.99 - 1.03)	0.240	1.01 (0.99 -1.03)
Birth_year2015:High_education_motherNo	1.01 (0.99 - 1.02)	0.407	1.01 (0.99 - 1.02)	1.01 (0.99 - 1.03)	0.271	1.01 (0.99 -1.02)
Birth_year2016:High_education_motherNo	1.00 (0.99 - 1.02)	0.736	1.00 (0.99 - 1.02)	1.01 (0.99 - 1.02)	0.352	1.01 (0.99 -1.02)
Birth_year2017:High_education_motherNo	1.00 (0.98 - 1.02)	0.948	1.00 (0.98 - 1.02)	1.00 (0.99 - 1.02)	0.621	1.00 (0.99 -1.02)
Birth_year2018:High_education_motherNo	1.00 (0.98 - 1.01)	0.896	1.00 (0.98 - 1.01)	1.01 (0.99 - 1.02)	0.508	1.01 (0.99 -1.02)
Birth_year2019:High_education_motherNo	1.00 (0.98 - 1.01)	0.793	1.00 (0.98 - 1.01)	1.00 (0.99 - 1.02)	0.646	1.00 (0.99 -1.02)
Birth_year2020:High_education_motherNo	0.99 (0.97 - 1.00)	0.148	0.99 (0.97 - 1.01)	1.00 (0.98 - 1.01)	0.624	1.00 (0.98 -1.01)
Birth_year2009:SDIhh_quartileFirst quartile	1.00 (0.97 - 1.02)	0.791	1.00 (0.97 - 1.02)	1.00 (0.97 - 1.02)	0.701	1.00 (0.97 -1.02)
Birth_year2010:SDIhh_quartileFirst quartile	1.00 (0.97 - 1.02)	0.724	1.00 (0.97 - 1.02)	1.00 (0.97 - 1.02)	0.681	1.00 (0.97 -1.02)
Birth_year2011:SDIhh_quartileFirst quartile	0.99 (0.97 - 1.02)	0.622	0.99 (0.97 - 1.02)	1.00 (0.97 - 1.02)	0.758	1.00 (0.97 -1.02)
Birth_year2012:SDIhh_quartileFirst quartile	1.00 (0.97 - 1.02)	0.768	1.00 (0.97 - 1.02)	1.00 (0.97 - 1.02)	0.858	1.00 (0.97 -1.02)
Birth_year2013:SDIhh_quartileFirst quartile	0.99 (0.97 - 1.02)	0.490	0.99 (0.97 - 1.02)	0.99 (0.97 - 1.02)	0.497	0.99 (0.97 -1.02)
Birth_year2014:SDIhh_quartileFirst quartile	0.99 (0.97 - 1.02)	0.444	0.99 (0.97 - 1.01)	0.99 (0.97 - 1.02)	0.505	0.99 (0.97 -1.02)
Birth_year2015:SDIhh_quartileFirst quartile	0.98 (0.96 - 1.01)	0.156	0.98 (0.96 - 1.01)	0.98 (0.96 - 1.01)	0.212	0.99 (0.96 -1.01)
Birth_year2016:SDIhh_quartileFirst quartile	0.97 (0.95 - 1.00)	0.037	0.97 (0.95 - 1.00)	0.98 (0.95 - 1.00)	0.076	0.98 (0.95 -1.00)
Birth_year2017:SDIhh_quartileFirst quartile	0.98 (0.95 - 1.00)	0.072	0.98 (0.95 - 1.00)	0.98 (0.95 - 1.00)	0.053	0.98 (0.95 -1.00)
Birth_year2018:SDIhh_quartileFirst quartile	0.98 (0.96 - 1.01)	0.134	0.98 (0.96 - 1.01)	0.98 (0.96 - 1.01)	0.128	0.98 (0.96 -1.01)
Birth_year2019:SDIhh_quartileFirst quartile	0.96 (0.94 - 0.99)	0.007	0.97 (0.94 - 0.99)	0.97 (0.95 - 1.00)	0.027	0.97 (0.95 -1.00)
Birth_year2020:SDIhh_quartileFirst quartile	0.96 (0.93 - 0.98)	0.001	0.96 (0.93 - 0.98)	0.96 (0.94 - 0.99)	0.003	0.96 (0.93 -0.99)
Birth_year2009:SDIhh_quartileSecond quartile	1.00 (0.98 - 1.02)	0.962	1.00 (0.98 - 1.02)	1.00 (0.98 - 1.02)	0.946	1.00 (0.98 -1.02)

Birth_year2010:SDIhh_quartileSecond quartile	1.00 (0.98 - 1.01)	0.643	1.00 (0.98 - 1.01)	0.99 (0.97 - 1.01)	0.500	0.99 (0.97 -1.01)
Birth_year2011:SDIhh_quartileSecond quartile	1.00 (0.98 - 1.02)	0.668	1.00 (0.98 - 1.02)	0.99 (0.97 - 1.01)	0.546	0.99 (0.97 -1.01)
Birth_year2012:SDIhh_quartileSecond quartile	1.00 (0.98 - 1.02)	0.652	1.00 (0.98 - 1.01)	0.99 (0.97 - 1.01)	0.505	0.99 (0.97 -1.01)
Birth_year2013:SDIhh_quartileSecond quartile	0.99 (0.97 - 1.01)	0.311	0.99 (0.97 - 1.01)	0.99 (0.97 - 1.01)	0.277	0.99 (0.97 -1.01)
Birth_year2014:SDIhh_quartileSecond quartile	0.99 (0.97 - 1.01)	0.184	0.99 (0.97 - 1.01)	0.99 (0.97 - 1.01)	0.185	0.99 (0.97 -1.01)
Birth_year2015:SDIhh_quartileSecond quartile	0.98 (0.96 - 1.00)	0.062	0.98 (0.96 - 1.00)	0.98 (0.96 - 1.00)	0.067	0.98 (0.96 -1.00)
Birth_year2016:SDIhh_quartileSecond quartile	0.98 (0.96 - 1.00)	0.016	0.98 (0.96 - 1.00)	0.98 (0.96 - 1.00)	0.014	0.98 (0.96 -1.00)
Birth_year2017:SDIhh_quartileSecond quartile	0.98 (0.96 - 1.00)	0.035	0.98 (0.96 - 1.00)	0.98 (0.96 - 1.00)	0.029	0.98 (0.96 -1.00)
Birth_year2018:SDIhh_quartileSecond quartile	0.98 (0.96 - 1.00)	0.036	0.98 (0.96 - 1.00)	0.97 (0.95 - 0.99)	0.011	0.97 (0.95 -0.99)
Birth_year2019:SDIhh_quartileSecond quartile	0.97 (0.95 - 0.99)	0.007	0.97 (0.95 - 0.99)	0.97 (0.95 - 0.99)	0.008	0.97 (0.95 -0.99)
Birth_year2020:SDIhh_quartileSecond quartile	0.97 (0.95 - 0.99)	0.001	0.97 (0.94 - 0.99)	0.97 (0.95 - 0.99)	0.002	0.97 (0.95 -0.99)
Birth_year2009:SDIhh_quartileThird quartile	1.00 (0.99 - 1.02)	0.692	1.00 (0.99 - 1.02)	1.00 (0.98 - 1.02)	0.911	1.00 (0.98 -1.02)
Birth_year2010:SDIhh_quartileThird quartile	1.00 (0.98 - 1.02)	0.921	1.00 (0.98 - 1.02)	1.00 (0.98 - 1.02)	0.886	1.00 (0.98 -1.02)
Birth_year2011:SDIhh_quartileThird quartile	1.00 (0.98 - 1.02)	0.784	1.00 (0.98 - 1.02)	1.00 (0.98 - 1.02)	0.974	1.00 (0.98 -1.02)
Birth_year2012:SDIhh_quartileThird quartile	1.00 (0.98 - 1.02)	0.840	1.00 (0.98 - 1.02)	1.00 (0.98 - 1.02)	0.877	1.00 (0.98 -1.02)
Birth_year2013:SDIhh_quartileThird quartile	1.00 (0.98 - 1.02)	1.000	1.00 (0.98 - 1.02)	1.00 (0.98 - 1.02)	0.827	1.00 (0.98 -1.02)
Birth_year2014:SDIhh_quartileThird quartile	1.00 (0.98 - 1.02)	0.908	1.00 (0.98 - 1.02)	1.00 (0.98 - 1.01)	0.686	1.00 (0.98 -1.02)
Birth_year2015:SDIhh_quartileThird quartile	0.99 (0.97 - 1.01)	0.460	0.99 (0.97 - 1.01)	0.99 (0.97 - 1.01)	0.353	0.99 (0.97 -1.01)
Birth_year2016:SDIhh_quartileThird quartile	0.99 (0.97 - 1.01)	0.440	0.99 (0.97 - 1.01)	0.99 (0.97 - 1.01)	0.290	0.99 (0.97 -1.01)
Birth_year2017:SDIhh_quartileThird quartile	0.99 (0.98 - 1.01)	0.548	0.99 (0.98 - 1.01)	0.99 (0.97 - 1.01)	0.352	0.99 (0.97 -1.01)
Birth_year2018:SDIhh_quartileThird quartile	1.00 (0.98 - 1.02)	0.721	1.00 (0.98 - 1.02)	0.99 (0.98 - 1.01)	0.503	0.99 (0.98 -1.01)
Birth_year2019:SDIhh_quartileThird quartile	0.99 (0.98 - 1.01)	0.541	0.99 (0.98 - 1.01)	0.99 (0.97 - 1.01)	0.451	0.99 (0.97 -1.01)
Birth_year2020:SDIhh_quartileThird quartile	0.99 (0.98 - 1.01)	0.577	0.99 (0.98 - 1.01)	0.99 (0.97 - 1.01)	0.415	0.99 (0.97 -1.01)
Birth_year2009:SDIhh_quartileUnknown	NA	NA	1.03 (0.94 - 1.11)	NA	NA	1.03 (0.94 -1.12)
Birth_year2010:SDIhh_quartileUnknown	NA	NA	1.04 (0.95 - 1.12)	NA	NA	1.04 (0.95 -1.13)
Birth_year2011:SDIhh_quartileUnknown	NA	NA	1.03 (0.95 - 1.12)	NA	NA	1.03 (0.94 -1.12)
Birth_year2012:SDIhh_quartileUnknown	NA	NA	1.06 (0.97 - 1.14)	NA	NA	1.06 (0.97 -1.15)
Birth_year2013:SDIhh_quartileUnknown	NA	NA	1.02 (0.93 - 1.11)	NA	NA	1.06 (0.96 -1.15)
Birth_year2014:SDIhh_quartileUnknown	NA	NA	1.03 (0.94 - 1.12)	NA	NA	1.07 (0.98 -1.17)
Birth_year2015:SDIhh_quartileUnknown	NA	NA	1.03 (0.94 - 1.12)	NA	NA	1.06 (0.96 -1.15)
Birth_year2016:SDIhh_quartileUnknown	NA	NA	1.00 (0.91 - 1.10)	NA	NA	1.03 (0.94 -1.13)
Birth_year2017:SDIhh_quartileUnknown	NA	NA	1.02 (0.92 - 1.11)	NA	NA	1.08 (0.98 -1.17)
Birth_year2018:SDIhh_quartileUnknown	NA	NA	1.04 (0.94 - 1.13)	NA	NA	1.09 (0.99 -1.19)
Birth_year2019:SDIhh_quartileUnknown	NA	NA	1.02 (0.93 - 1.12)	NA	NA	1.09 (0.99 -1.18)
Birth_year2020:SDIhh_quartileUnknown	NA	NA	0.93 (0.84 - 1.03)	NA	NA	1.03 (0.93 -1.12)

Birth_year2009:Country_origin_thirdgenEurope (excl NL)	1.00 (0.98 - 1.03)	0.893	1.00 (0.98 - 1.03)	1.00 (0.98 - 1.02)	0.944	1.00 (0.98 -1.03)
Birth_year2010:Country_origin_thirdgenEurope (excl NL)	1.00 (0.98 - 1.03)	0.873	1.00 (0.98 - 1.02)	1.00 (0.98 - 1.03)	0.678	1.01 (0.98 -1.03)
Birth_year2011:Country_origin_thirdgenEurope (excl NL)	1.00 (0.98 - 1.03)	0.758	1.00 (0.98 - 1.02)	1.00 (0.98 - 1.03)	0.689	1.01 (0.98 -1.03)
Birth_year2012:Country_origin_thirdgenEurope (excl NL)	1.00 (0.98 - 1.03)	0.758	1.00 (0.98 - 1.03)	1.00 (0.98 - 1.03)	0.774	1.01 (0.98 -1.03)
Birth_year2013:Country_origin_thirdgenEurope (excl NL)	1.00 (0.98 - 1.03)	0.884	1.00 (0.98 - 1.02)	1.00 (0.98 - 1.03)	0.755	1.01 (0.98 -1.03)
Birth_year2014:Country_origin_thirdgenEurope (excl NL)	1.00 (0.98 - 1.03)	0.816	1.00 (0.98 - 1.02)	1.00 (0.98 - 1.03)	0.719	1.01 (0.98 -1.03)
Birth_year2015:Country_origin_thirdgenEurope (excl NL)	1.00 (0.98 - 1.02)	0.983	1.00 (0.98 - 1.02)	1.01 (0.98 - 1.03)	0.646	1.01 (0.99 -1.03)
Birth_year2016:Country_origin_thirdgenEurope (excl NL)	1.00 (0.97 - 1.02)	0.780	1.00 (0.97 - 1.02)	1.00 (0.98 - 1.03)	0.745	1.01 (0.99 -1.03)
Birth_year2017:Country_origin_thirdgenEurope (excl NL)	1.00 (0.98 - 1.02)	0.935	1.00 (0.98 - 1.02)	1.00 (0.98 - 1.03)	0.831	1.01 (0.99 -1.03)
Birth_year2018:Country_origin_thirdgenEurope (excl NL)	1.00 (0.98 - 1.02)	0.999	1.00 (0.98 - 1.03)	1.00 (0.98 - 1.03)	0.872	1.01 (0.99 -1.04)
Birth_year2019:Country_origin_thirdgenEurope (excl NL)	1.00 (0.97 - 1.02)	0.805	1.00 (0.98 - 1.02)	1.00 (0.98 - 1.03)	0.816	1.02 (0.99 -1.04)
Birth_year2020:Country_origin_thirdgenEurope (excl NL)	0.99 (0.97 - 1.02)	0.503	1.00 (0.98 - 1.02)	1.00 (0.98 - 1.02)	0.954	1.01 (0.99 -1.04)
Birth_year2009:Country_origin_thirdgenIndonesia	1.00 (0.97 - 1.04)	0.915	1.00 (0.97 - 1.04)	1.01 (0.97 - 1.04)	0.704	1.01 (0.97 -1.04)
Birth_year2010:Country_origin_thirdgenIndonesia	1.00 (0.97 - 1.04)	0.894	1.00 (0.97 - 1.04)	1.01 (0.97 - 1.04)	0.685	1.01 (0.97 -1.04)
Birth_year2011:Country_origin_thirdgenIndonesia	1.00 (0.97 - 1.03)	0.971	1.00 (0.97 - 1.03)	1.00 (0.97 - 1.04)	0.994	1.00 (0.97 -1.03)
Birth_year2012:Country_origin_thirdgenIndonesia	1.00 (0.97 - 1.04)	0.834	1.00 (0.97 - 1.04)	1.01 (0.97 - 1.04)	0.683	1.01 (0.97 -1.04)
Birth_year2013:Country_origin_thirdgenIndonesia	1.00 (0.97 - 1.04)	0.877	1.00 (0.97 - 1.04)	1.01 (0.97 - 1.04)	0.771	1.00 (0.97 -1.04)
Birth_year2014:Country_origin_thirdgenIndonesia	1.00 (0.97 - 1.04)	0.917	1.00 (0.97 - 1.04)	1.01 (0.97 - 1.04)	0.737	1.00 (0.97 -1.04)
Birth_year2015:Country_origin_thirdgenIndonesia	1.00 (0.97 - 1.04)	0.965	1.00 (0.96 - 1.03)	1.00 (0.97 - 1.04)	0.941	1.00 (0.96 -1.03)
Birth_year2016:Country_origin_thirdgenIndonesia	1.00 (0.96 - 1.04)	0.956	1.00 (0.96 - 1.04)	1.00 (0.97 - 1.04)	0.882	1.00 (0.97 -1.04)
Birth_year2017:Country_origin_thirdgenIndonesia	1.00 (0.97 - 1.04)	0.896	1.00 (0.97 - 1.04)	1.01 (0.97 - 1.04)	0.787	1.01 (0.97 -1.04)
Birth_year2018:Country_origin_thirdgenIndonesia	1.00 (0.96 - 1.04)	0.970	1.00 (0.96 - 1.04)	1.00 (0.96 - 1.04)	0.929	1.00 (0.97 -1.04)
Birth_year2019:Country_origin_thirdgenIndonesia	0.99 (0.95 - 1.03)	0.576	0.99 (0.95 - 1.03)	1.00 (0.96 - 1.04)	0.973	1.00 (0.96 -1.04)
Birth_year2020:Country_origin_thirdgenIndonesia	0.99 (0.95 - 1.03)	0.563	0.99 (0.95 - 1.03)	0.99 (0.95 - 1.03)	0.582	0.99 (0.95 -1.03)
Birth_year2009:Country_origin_thirdgenMorocco	1.00 (0.96 - 1.03)	0.800	1.00 (0.96 - 1.03)	1.00 (0.96 - 1.03)	0.889	1.00 (0.97 -1.03)

Birth_year2010:Country_origin_thirdgenMorocco	1.00 (0.97 - 1.04)	0.853	1.00 (0.97 - 1.04)	1.01 (0.97 - 1.04)	0.728	1.01 (0.97 -1.04)
Birth_year2011:Country_origin_thirdgenMorocco	1.00 (0.96 - 1.03)	0.855	1.00 (0.97 - 1.03)	1.00 (0.97 - 1.04)	0.877	1.00 (0.97 -1.04)
Birth_year2012:Country_origin_thirdgenMorocco	1.00 (0.96 - 1.03)	0.829	1.00 (0.96 - 1.03)	1.00 (0.97 - 1.04)	0.840	1.01 (0.97 -1.04)
Birth_year2013:Country_origin_thirdgenMorocco	0.99 (0.95 - 1.02)	0.403	0.99 (0.95 - 1.02)	0.99 (0.96 - 1.03)	0.726	1.00 (0.96 -1.03)
Birth_year2014:Country_origin_thirdgenMorocco	0.97 (0.94 - 1.00)	0.071	0.97 (0.93 - 1.00)	0.98 (0.95 - 1.02)	0.348	0.99 (0.95 -1.02)
Birth_year2015:Country_origin_thirdgenMorocco	0.93 (0.90 - 0.96)	<0.001	0.93 (0.90 - 0.97)	0.95 (0.92 - 0.98)	0.003	0.95 (0.92 -0.98)
Birth_year2016:Country_origin_thirdgenMorocco	0.91 (0.87 - 0.94)	<0.001	0.90 (0.87 - 0.94)	0.92 (0.89 - 0.95)	<0.001	0.92 (0.89 -0.96)
Birth_year2017:Country_origin_thirdgenMorocco	0.90 (0.87 - 0.93)	<0.001	0.90 (0.87 - 0.94)	0.91 (0.88 - 0.94)	<0.001	0.91 (0.88 -0.95)
Birth_year2018:Country_origin_thirdgenMorocco	0.87 (0.84 - 0.90)	<0.001	0.87 (0.84 - 0.91)	0.88 (0.85 - 0.92)	<0.001	0.89 (0.85 -0.92)
Birth_year2019:Country_origin_thirdgenMorocco	0.79 (0.76 - 0.82)	<0.001	0.79 (0.76 - 0.83)	0.83 (0.80 - 0.86)	<0.001	0.83 (0.79 -0.86)
Birth_year2020:Country_origin_thirdgenMorocco	0.73 (0.70 - 0.75)	<0.001	0.73 (0.69 - 0.77)	0.75 (0.72 - 0.78)	<0.001	0.75 (0.71 -0.79)
Birth_year2009:Country_origin_thirdgenOther, Africa	1.01 (0.97 - 1.05)	0.684	1.01 (0.97 - 1.05)	1.01 (0.97 - 1.06)	0.586	1.01 (0.96 -1.05)
Birth_year2010:Country_origin_thirdgenOther, Africa	1.02 (0.98 - 1.06)	0.373	1.03 (0.99 - 1.07)	1.03 (0.99 - 1.07)	0.202	1.03 (0.99 -1.07)
Birth_year2011:Country_origin_thirdgenOther, Africa	1.03 (0.99 - 1.08)	0.142	1.04 (1.00 - 1.08)	1.05 (1.00 - 1.09)	0.032	1.05 (1.01 -1.10)
Birth_year2012:Country_origin_thirdgenOther, Africa	1.03 (0.98 - 1.07)	0.222	1.04 (1.00 - 1.08)	1.04 (1.00 - 1.09)	0.057	1.05 (1.01 -1.09)
Birth_year2013:Country_origin_thirdgenOther, Africa	1.04 (0.99 - 1.08)	0.093	1.05 (1.01 - 1.09)	1.04 (1.00 - 1.09)	0.042	1.05 (1.01 -1.09)
Birth_year2014:Country_origin_thirdgenOther, Africa	1.02 (0.98 - 1.07)	0.306	1.04 (1.00 - 1.08)	1.04 (1.00 - 1.09)	0.054	1.05 (1.01 -1.09)
Birth_year2015:Country_origin_thirdgenOther, Africa	1.03 (0.99 - 1.08)	0.134	1.04 (1.00 - 1.08)	1.05 (1.01 - 1.10)	0.022	1.05 (1.01 -1.09)
Birth_year2016:Country_origin_thirdgenOther, Africa	1.03 (0.99 - 1.08)	0.146	1.04 (1.00 - 1.08)	1.05 (1.01 - 1.09)	0.025	1.05 (1.01 -1.09)
Birth_year2017:Country_origin_thirdgenOther, Africa	1.03 (0.99 - 1.07)	0.183	1.04 (1.00 - 1.08)	1.05 (1.01 - 1.09)	0.021	1.05 (1.01 -1.09)
Birth_year2018:Country_origin_thirdgenOther, Africa	1.03 (0.99 - 1.07)	0.126	1.04 (1.00 - 1.08)	1.05 (1.01 - 1.09)	0.020	1.05 (1.01 -1.09)
Birth_year2019:Country_origin_thirdgenOther, Africa	1.02 (0.98 - 1.06)	0.325	1.03 (0.99 - 1.07)	1.04 (1.00 - 1.08)	0.050	1.04 (1.00 -1.08)
Birth_year2020:Country_origin_thirdgenOther, Africa	1.01 (0.97 - 1.05)	0.707	1.02 (0.98 - 1.06)	1.04 (1.00 - 1.08)	0.071	1.03 (0.99 -1.07)
Birth_year2009:Country_origin_thirdgenOther, America/Oceania	1.00 (0.95 - 1.05)	0.907	1.00 (0.95 - 1.04)	0.99 (0.94 - 1.04)	0.694	1.00 (0.95 -1.05)
Birth_year2010:Country_origin_thirdgenOther, America/Oceania	1.00 (0.96 - 1.05)	0.892	1.00 (0.95 - 1.04)	1.00 (0.95 - 1.05)	0.944	1.00 (0.96 -1.05)
Birth_year2011:Country_origin_thirdgenOther, America/Oceania	1.00 (0.96 - 1.05)	0.920	1.00 (0.96 - 1.05)	1.00 (0.96 - 1.05)	0.882	1.01 (0.97 -1.06)
Birth_year2012:Country_origin_thirdgenOther, America/Oceania	1.00 (0.95 - 1.05)	0.988	1.00 (0.95 - 1.04)	0.99 (0.95 - 1.04)	0.794	1.00 (0.95 -1.04)
Birth_year2013:Country_origin_thirdgenOther, America/Oceania	1.00 (0.96 - 1.05)	0.839	1.00 (0.95 - 1.04)	1.00 (0.96 - 1.05)	0.918	1.01 (0.96 -1.06)
Birth_year2014:Country_origin_thirdgenOther, America/Oceania	1.00 (0.96 - 1.05)	0.912	1.00 (0.96 - 1.04)	1.00 (0.96 - 1.05)	0.855	1.02 (0.97 -1.06)
Birth_year2015:Country_origin_thirdgenOther, America/Oceania	1.00 (0.96 - 1.05)	0.938	1.00 (0.95 - 1.04)	1.01 (0.96 - 1.05)	0.828	1.02 (0.97 -1.06)

Birth_year2016:Country_origin_thirdgenOther, America/Oceania	1.01 (0.96 - 1.06)	0.782	1.00 (0.96 - 1.04)	1.01 (0.96 - 1.06)	0.654	1.03 (0.99 -1.08)
Birth_year2017:Country_origin_thirdgenOther, America/Oceania	1.01 (0.96 - 1.06)	0.711	1.00 (0.96 - 1.05)	1.01 (0.96 - 1.05)	0.806	1.03 (0.98 -1.07)
Birth_year2018:Country_origin_thirdgenOther, America/Oceania	1.01 (0.96 - 1.06)	0.717	1.00 (0.96 - 1.04)	1.01 (0.97 - 1.06)	0.578	1.04 (0.99 -1.08)
Birth_year2019:Country_origin_thirdgenOther, America/Oceania	1.00 (0.96 - 1.05)	0.921	0.99 (0.95 - 1.04)	1.01 (0.96 - 1.05)	0.777	1.03 (0.99 -1.07)
Birth_year2020:Country_origin_thirdgenOther, America/Oceania	1.00 (0.95 - 1.05)	0.997	0.99 (0.95 - 1.04)	1.00 (0.96 - 1.05)	0.874	1.03 (0.99 -1.07)
Birth_year2009:Country_origin_thirdgenOther, Asia	1.00 (0.97 - 1.04)	0.887	1.00 (0.97 - 1.04)	1.01 (0.97 - 1.05)	0.623	1.00 (0.97 -1.04)
Birth_year2010:Country_origin_thirdgenOther, Asia	1.01 (0.98 - 1.05)	0.443	1.02 (0.98 - 1.05)	1.02 (0.98 - 1.06)	0.285	1.02 (0.99 -1.06)
Birth_year2011:Country_origin_thirdgenOther, Asia	1.01 (0.98 - 1.05)	0.445	1.02 (0.98 - 1.05)	1.02 (0.99 - 1.06)	0.180	1.02 (0.99 -1.06)
Birth_year2012:Country_origin_thirdgenOther, Asia	1.01 (0.98 - 1.05)	0.487	1.02 (0.98 - 1.05)	1.03 (0.99 - 1.06)	0.150	1.03 (1.00 -1.06)
Birth_year2013:Country_origin_thirdgenOther, Asia	1.01 (0.98 - 1.05)	0.434	1.02 (0.99 - 1.06)	1.03 (0.99 - 1.06)	0.137	1.03 (1.00 -1.07)
Birth_year2014:Country_origin_thirdgenOther, Asia	1.01 (0.97 - 1.04)	0.611	1.02 (0.98 - 1.05)	1.02 (0.99 - 1.06)	0.188	1.03 (1.00 -1.06)
Birth_year2015:Country_origin_thirdgenOther, Asia	0.99 (0.96 - 1.03)	0.726	1.01 (0.97 - 1.04)	1.01 (0.98 - 1.05)	0.495	1.02 (0.99 -1.06)
Birth_year2016:Country_origin_thirdgenOther, Asia	1.01 (0.98 - 1.05)	0.499	1.02 (0.99 - 1.05)	1.03 (0.99 - 1.06)	0.105	1.03 (1.00 -1.07)
Birth_year2017:Country_origin_thirdgenOther, Asia	1.02 (0.99 - 1.06)	0.186	1.03 (1.00 - 1.07)	1.04 (1.01 - 1.08)	0.020	1.05 (1.02 -1.08)
Birth_year2018:Country_origin_thirdgenOther, Asia	1.02 (0.99 - 1.06)	0.169	1.03 (1.00 - 1.07)	1.04 (1.01 - 1.08)	0.017	1.05 (1.02 -1.08)
Birth_year2019:Country_origin_thirdgenOther, Asia	1.03 (0.99 - 1.06)	0.134	1.04 (1.01 - 1.07)	1.04 (1.01 - 1.08)	0.014	1.05 (1.02 -1.08)
Birth_year2020:Country_origin_thirdgenOther, Asia	1.03 (0.99 - 1.06)	0.123	1.04 (1.01 - 1.07)	1.04 (1.01 - 1.08)	0.014	1.05 (1.02 -1.08)
Birth_year2009:Country_origin_thirdgenSuriname	1.00 (0.96 - 1.04)	0.992	1.00 (0.96 - 1.04)	1.00 (0.97 - 1.04)	0.863	1.01 (0.97 -1.04)
Birth_year2010:Country_origin_thirdgenSuriname	1.00 (0.97 - 1.04)	0.866	1.00 (0.97 - 1.04)	1.01 (0.97 - 1.05)	0.633	1.01 (0.97 -1.05)
Birth_year2011:Country_origin_thirdgenSuriname	1.00 (0.96 - 1.04)	0.887	1.00 (0.97 - 1.04)	1.01 (0.97 - 1.05)	0.546	1.02 (0.98 -1.05)
Birth_year2012:Country_origin_thirdgenSuriname	1.01 (0.97 - 1.05)	0.754	1.01 (0.97 - 1.04)	1.01 (0.98 - 1.06)	0.458	1.02 (0.98 -1.06)
Birth_year2013:Country_origin_thirdgenSuriname	1.00 (0.97 - 1.04)	0.835	1.00 (0.96 - 1.04)	1.01 (0.97 - 1.05)	0.514	1.02 (0.98 -1.05)
Birth_year2014:Country_origin_thirdgenSuriname	1.01 (0.97 - 1.05)	0.665	1.01 (0.97 - 1.05)	1.02 (0.98 - 1.06)	0.420	1.02 (0.98 -1.06)
Birth_year2015:Country_origin_thirdgenSuriname	1.00 (0.96 - 1.04)	0.826	1.00 (0.96 - 1.03)	1.01 (0.97 - 1.05)	0.570	1.01 (0.97 -1.05)
Birth_year2016:Country_origin_thirdgenSuriname	0.98 (0.94 - 1.02)	0.313	0.98 (0.94 - 1.02)	0.99 (0.96 - 1.03)	0.739	0.99 (0.95 -1.03)
Birth_year2017:Country_origin_thirdgenSuriname	0.99 (0.95 - 1.03)	0.517	0.99 (0.95 - 1.03)	1.00 (0.96 - 1.04)	0.912	1.00 (0.96 -1.04)
Birth_year2018:Country_origin_thirdgenSuriname	0.97 (0.94 - 1.01)	0.177	0.97 (0.93 - 1.01)	0.99 (0.95 - 1.03)	0.515	0.99 (0.95 -1.03)
Birth_year2019:Country_origin_thirdgenSuriname	0.95 (0.92 - 0.99)	0.020	0.95 (0.91 - 0.99)	0.98 (0.94 - 1.01)	0.216	0.97 (0.93 -1.01)
Birth_year2020:Country_origin_thirdgenSuriname	0.93 (0.90 - 0.97)	0.001	0.93 (0.89 - 0.97)	0.95 (0.92 - 0.99)	0.024	0.95 (0.91 -0.99)
Birth_year2009:Country_origin_thirdgenThe Dutch Caribbean	1.01 (0.96 - 1.06)	0.772	1.01 (0.96 - 1.06)	1.01 (0.96 - 1.07)	0.596	1.02 (0.97 -1.07)

Birth_year2010:Country_origin_thirdgenThe Dutch Caribbean	1.00 (0.95 - 1.06)	0.852	1.01 (0.96 - 1.06)	1.01 (0.96 - 1.06)	0.704	1.01 (0.96 -1.06)
Birth_year2011:Country_origin_thirdgenThe Dutch Caribbean	1.01 (0.96 - 1.06)	0.727	1.01 (0.96 - 1.06)	1.01 (0.96 - 1.07)	0.595	1.02 (0.97 -1.07)
Birth_year2012:Country_origin_thirdgenThe Dutch Caribbean	1.01 (0.96 - 1.06)	0.817	1.01 (0.96 - 1.06)	1.01 (0.96 - 1.07)	0.642	1.01 (0.96 -1.06)
Birth_year2013:Country_origin_thirdgenThe Dutch Caribbean	1.01 (0.96 - 1.06)	0.733	1.01 (0.96 - 1.06)	1.02 (0.97 - 1.08)	0.431	1.02 (0.97 -1.07)
Birth_year2014:Country_origin_thirdgenThe Dutch Caribbean	1.01 (0.96 - 1.06)	0.708	1.01 (0.96 - 1.06)	1.02 (0.97 - 1.08)	0.387	1.01 (0.96 -1.06)
Birth_year2015:Country_origin_thirdgenThe Dutch Caribbean	1.00 (0.95 - 1.06)	0.892	1.00 (0.95 - 1.05)	1.02 (0.97 - 1.07)	0.499	1.01 (0.96 -1.06)
Birth_year2016:Country_origin_thirdgenThe Dutch Caribbean	0.98 (0.93 - 1.03)	0.506	0.98 (0.93 - 1.03)	1.00 (0.95 - 1.05)	0.937	0.99 (0.94 -1.04)
Birth_year2017:Country_origin_thirdgenThe Dutch Caribbean	0.99 (0.94 - 1.04)	0.673	0.99 (0.94 - 1.04)	1.00 (0.95 - 1.05)	0.980	0.99 (0.94 -1.04)
Birth_year2018:Country_origin_thirdgenThe Dutch Caribbean	0.97 (0.92 - 1.02)	0.273	0.97 (0.92 - 1.02)	0.98 (0.94 - 1.04)	0.555	0.97 (0.92 -1.02)
Birth_year2019:Country_origin_thirdgenThe Dutch Caribbean	0.95 (0.90 - 1.00)	0.062	0.95 (0.90 - 1.00)	0.97 (0.92 - 1.02)	0.268	0.96 (0.91 -1.01)
Birth_year2020:Country_origin_thirdgenThe Dutch Caribbean	0.93 (0.88 - 0.98)	0.004	0.93 (0.88 - 0.98)	0.95 (0.91 - 1.00)	0.071	0.94 (0.89 -0.99)
Birth_year2009:Country_origin_thirdgenTurkey	1.01 (0.97 - 1.04)	0.787	1.00 (0.97 - 1.04)	1.01 (0.97 - 1.05)	0.617	1.01 (0.97 -1.05)
Birth_year2010:Country_origin_thirdgenTurkey	1.01 (0.98 - 1.05)	0.532	1.01 (0.98 - 1.05)	1.02 (0.98 - 1.06)	0.385	1.02 (0.98 -1.05)
Birth_year2011:Country_origin_thirdgenTurkey	1.01 (0.97 - 1.05)	0.585	1.01 (0.98 - 1.05)	1.02 (0.98 - 1.05)	0.394	1.02 (0.98 -1.06)
Birth_year2012:Country_origin_thirdgenTurkey	1.01 (0.98 - 1.05)	0.509	1.02 (0.98 - 1.05)	1.02 (0.98 - 1.06)	0.290	1.03 (0.99 -1.07)
Birth_year2013:Country_origin_thirdgenTurkey	1.02 (0.98 - 1.05)	0.431	1.02 (0.98 - 1.05)	1.02 (0.98 - 1.06)	0.304	1.02 (0.99 -1.06)
Birth_year2014:Country_origin_thirdgenTurkey	1.01 (0.98 - 1.05)	0.510	1.01 (0.98 - 1.05)	1.02 (0.98 - 1.06)	0.345	1.02 (0.98 -1.06)
Birth_year2015:Country_origin_thirdgenTurkey	1.00 (0.96 - 1.03)	0.844	1.00 (0.96 - 1.03)	1.01 (0.97 - 1.05)	0.611	1.01 (0.98 -1.05)
Birth_year2016:Country_origin_thirdgenTurkey	0.98 (0.94 - 1.01)	0.182	0.98 (0.94 - 1.01)	0.99 (0.96 - 1.03)	0.656	0.99 (0.96 -1.03)
Birth_year2017:Country_origin_thirdgenTurkey	0.97 (0.93 - 1.00)	0.067	0.97 (0.93 - 1.00)	0.98 (0.95 - 1.02)	0.336	0.98 (0.94 -1.02)
Birth_year2018:Country_origin_thirdgenTurkey	0.96 (0.92 - 0.99)	0.019	0.96 (0.92 - 0.99)	0.97 (0.94 - 1.01)	0.173	0.97 (0.94 -1.01)
Birth_year2019:Country_origin_thirdgenTurkey	0.90 (0.87 - 0.94)	<0.001	0.90 (0.87 - 0.94)	0.94 (0.90 - 0.97)	0.001	0.94 (0.90 -0.97)
Birth_year2020:Country_origin_thirdgenTurkey	0.87 (0.84 - 0.91)	<0.001	0.87 (0.83 - 0.91)	0.90 (0.86 - 0.93)	<0.001	0.90 (0.86 -0.94)
Birth_year2009:SES.mother5Self-employed	0.99 (0.97 - 1.02)	0.667	0.99 (0.97 - 1.02)	0.99 (0.97 - 1.02)	0.564	0.99 (0.97 -1.02)
Birth_year2010:SES.mother5Self-employed	0.99 (0.97 - 1.02)	0.607	0.99 (0.97 - 1.02)	0.99 (0.97 - 1.02)	0.511	0.99 (0.96 -1.02)
Birth_year2011:SES.mother5Self-employed	0.99 (0.97 - 1.02)	0.688	0.99 (0.97 - 1.02)	0.99 (0.96 - 1.02)	0.478	0.99 (0.96 -1.02)

Birth_year2012:SES.mother5Self-employed	0.99 (0.97 - 1.02)	0.515	0.99 (0.96 - 1.02)	0.99 (0.96 - 1.01)	0.286	0.99 (0.96 -1.01)
Birth_year2013:SES.mother5Self-employed	0.99 (0.96 - 1.01)	0.269	0.98 (0.96 - 1.01)	0.98 (0.95 - 1.01)	0.140	0.98 (0.95 -1.01)
Birth_year2014:SES.mother5Self-employed	0.98 (0.95 - 1.00)	0.097	0.98 (0.95 - 1.00)	0.97 (0.95 - 1.00)	0.059	0.97 (0.95 -1.00)
Birth_year2015:SES.mother5Self-employed	0.97 (0.95 - 1.00)	0.055	0.97 (0.95 - 1.00)	0.97 (0.95 - 1.00)	0.034	0.97 (0.95 -1.00)
Birth_year2016:SES.mother5Self-employed	0.97 (0.95 - 1.00)	0.033	0.97 (0.95 - 1.00)	0.97 (0.94 - 0.99)	0.012	0.97 (0.94 -0.99)
Birth_year2017:SES.mother5Self-employed	0.99 (0.96 - 1.01)	0.312	0.99 (0.96 - 1.01)	0.98 (0.95 - 1.00)	0.097	0.98 (0.95 -1.00)
Birth_year2018:SES.mother5Self-employed	0.98 (0.96 - 1.01)	0.193	0.98 (0.96 - 1.01)	0.97 (0.95 - 1.00)	0.057	0.97 (0.95 -1.00)
Birth_year2019:SES.mother5Self-employed	0.98 (0.95 - 1.01)	0.118	0.98 (0.95 - 1.01)	0.97 (0.95 - 1.00)	0.046	0.97 (0.95 -1.00)
Birth_year2020:SES.mother5Self-employed	0.97 (0.94 - 1.00)	0.021	0.97 (0.94 - 1.00)	0.96 (0.94 - 0.99)	0.006	0.96 (0.94 -0.99)
Birth_year2009:SES.mother5Recipient benefit	1.01 (0.98 - 1.03)	0.645	1.01 (0.98 - 1.03)	1.00 (0.98 - 1.03)	0.770	1.00 (0.98 -1.03)
Birth_year2010:SES.mother5Recipient benefit	1.01 (0.99 - 1.04)	0.291	1.01 (0.99 - 1.03)	1.01 (0.99 - 1.03)	0.382	1.01 (0.99 -1.03)
Birth_year2011:SES.mother5Recipient benefit	1.01 (0.99 - 1.03)	0.388	1.01 (0.99 - 1.03)	1.01 (0.98 - 1.03)	0.512	1.01 (0.98 -1.03)
Birth_year2012:SES.mother5Recipient benefit	1.01 (0.99 - 1.04)	0.265	1.01 (0.99 - 1.04)	1.01 (0.99 - 1.04)	0.258	1.01 (0.99 -1.04)
Birth_year2013:SES.mother5Recipient benefit	1.01 (0.99 - 1.03)	0.390	1.01 (0.99 - 1.03)	1.01 (0.99 - 1.04)	0.312	1.01 (0.99 -1.03)
Birth_year2014:SES.mother5Recipient benefit	1.00 (0.98 - 1.02)	0.988	1.00 (0.98 - 1.02)	1.01 (0.98 - 1.03)	0.508	1.01 (0.98 -1.03)
Birth_year2015:SES.mother5Recipient benefit	0.99 (0.96 - 1.01)	0.323	0.99 (0.96 - 1.01)	1.00 (0.97 - 1.02)	0.897	1.00 (0.97 -1.02)
Birth_year2016:SES.mother5Recipient benefit	1.00 (0.98 - 1.03)	0.950	1.00 (0.98 - 1.03)	1.01 (0.98 - 1.03)	0.537	1.01 (0.98 -1.03)
Birth_year2017:SES.mother5Recipient benefit	1.01 (0.99 - 1.04)	0.404	1.01 (0.99 - 1.03)	1.01 (0.99 - 1.04)	0.383	1.01 (0.99 -1.04)
Birth_year2018:SES.mother5Recipient benefit	1.01 (0.99 - 1.04)	0.234	1.02 (0.99 - 1.04)	1.02 (0.99 - 1.04)	0.136	1.02 (0.99 -1.04)
Birth_year2019:SES.mother5Recipient benefit	1.01 (0.99 - 1.04)	0.315	1.01 (0.99 - 1.04)	1.02 (0.99 - 1.05)	0.120	1.02 (1.00 -1.05)
Birth_year2020:SES.mother5Recipient benefit	1.01 (0.98 - 1.03)	0.548	1.01 (0.98 - 1.03)	1.01 (0.99 - 1.04)	0.342	1.01 (0.99 -1.04)
Birth_year2009:SES.mother5Recipient benefit	1.01 (0.94 - 1.09)	0.820	1.01 (0.93 - 1.08)	1.01 (0.94 - 1.09)	0.719	1.01 (0.94 -1.09)
Birth_year2010:SES.mother5Recipient benefit	1.01 (0.93 - 1.09)	0.807	1.01 (0.93 - 1.09)	1.01 (0.93 - 1.09)	0.872	1.01 (0.93 -1.08)
Birth_year2011:SES.mother5Recipient benefit	1.00 (0.92 - 1.09)	0.907	1.00 (0.92 - 1.09)	0.99 (0.92 - 1.08)	0.906	1.00 (0.91 -1.08)
Birth_year2012:SES.mother5Recipient benefit	1.00 (0.92 - 1.10)	0.966	1.00 (0.91 - 1.09)	0.99 (0.90 - 1.08)	0.820	0.99 (0.90 -1.08)
Birth_year2013:SES.mother5Recipient benefit	0.98 (0.90 - 1.07)	0.718	0.98 (0.90 - 1.07)	0.99 (0.91 - 1.08)	0.844	0.99 (0.90 -1.08)
Birth_year2014:SES.mother5Recipient benefit	0.99 (0.91 - 1.08)	0.864	0.99 (0.90 - 1.08)	0.99 (0.90 - 1.08)	0.783	0.99 (0.90 -1.08)
Birth_year2015:SES.mother5Recipient benefit	0.97 (0.88 - 1.07)	0.555	0.97 (0.87 - 1.07)	0.97 (0.88 - 1.08)	0.610	0.97 (0.87 -1.07)
Birth_year2016:SES.mother5Recipient benefit	1.00 (0.90 - 1.11)	0.994	1.00 (0.89 - 1.10)	0.99 (0.89 - 1.10)	0.791	0.98 (0.88 -1.09)
Birth_year2017:SES.mother5Recipient benefit	1.00 (0.89 - 1.11)	0.941	0.99 (0.88 - 1.10)	0.99 (0.89 - 1.10)	0.844	0.98 (0.87 -1.09)
Birth_year2018:SES.mother5Recipient benefit	1.01 (0.90 - 1.12)	0.913	1.01 (0.90 - 1.12)	1.00 (0.89 - 1.11)	0.935	1.00 (0.88 -1.11)
Birth_year2019:SES.mother5Recipient benefit	1.03 (0.90 - 1.17)	0.697	1.03 (0.90 - 1.16)	1.02 (0.89 - 1.16)	0.801	1.02 (0.89 -1.15)
Birth_year2020:SES.mother5Recipient benefit	0.98 (0.85 - 1.13)	0.786	0.98 (0.83 - 1.12)	0.97 (0.84 - 1.12)	0.639	0.97 (0.82 -1.11)
Birth_year2009:SES.mother5Child/student	1.00 (0.95 - 1.05)	0.977	1.00 (0.95 - 1.05)	0.99 (0.94 - 1.04)	0.735	0.99 (0.94 -1.04)
Birth_year2010:SES.mother5Child/student	1.01 (0.96 - 1.06)	0.810	1.00 (0.95 - 1.06)	1.00 (0.95 - 1.05)	0.982	1.00 (0.95 -1.05)
Birth_year2011:SES.mother5Child/student	1.01 (0.95 - 1.06)	0.846	1.00 (0.95 - 1.06)	1.00 (0.95 - 1.05)	0.945	1.00 (0.94 -1.05)

Birth_year2012:SES.mother5Child/student	1.00 (0.95 - 1.06)	0.910	1.00 (0.95 - 1.05)	0.99 (0.94 - 1.05)	0.803	0.99 (0.94 -1.05)
Birth_year2013:SES.mother5Child/student	1.01 (0.95 - 1.06)	0.824	1.00 (0.95 - 1.06)	1.01 (0.95 - 1.06)	0.791	1.01 (0.95 -1.06)
Birth_year2014:SES.mother5Child/student	0.99 (0.94 - 1.05)	0.731	0.99 (0.93 - 1.04)	0.99 (0.94 - 1.05)	0.795	0.99 (0.94 -1.05)
Birth_year2015:SES.mother5Child/student	0.97 (0.91 - 1.02)	0.227	0.96 (0.91 - 1.02)	0.97 (0.92 - 1.03)	0.318	0.97 (0.91 -1.03)
Birth_year2016:SES.mother5Child/student	0.97 (0.92 - 1.03)	0.333	0.97 (0.91 - 1.03)	0.97 (0.92 - 1.03)	0.378	0.97 (0.92 -1.03)
Birth_year2017:SES.mother5Child/student	0.99 (0.94 - 1.05)	0.737	0.99 (0.93 - 1.05)	0.99 (0.94 - 1.05)	0.792	0.99 (0.94 -1.05)
Birth_year2018:SES.mother5Child/student	0.99 (0.94 - 1.05)	0.744	0.99 (0.93 - 1.05)	0.98 (0.93 - 1.04)	0.598	0.99 (0.93 -1.04)
Birth_year2019:SES.mother5Child/student	0.98 (0.93 - 1.04)	0.531	0.98 (0.92 - 1.04)	0.98 (0.93 - 1.04)	0.548	0.98 (0.93 -1.04)
Birth_year2020:SES.mother5Child/student	0.96 (0.90 - 1.02)	0.157	0.96 (0.90 - 1.02)	0.97 (0.91 - 1.03)	0.274	0.97 (0.91 -1.03)
Birth_year2009:SES.mother5Other	1.00 (0.97 - 1.03)	0.973	1.00 (0.97 - 1.03)	1.00 (0.97 - 1.03)	0.941	1.00 (0.97 -1.02)
Birth_year2010:SES.mother5Other	1.00 (0.98 - 1.03)	0.926	1.00 (0.98 - 1.03)	1.00 (0.98 - 1.03)	0.935	1.00 (0.98 -1.03)
Birth_year2011:SES.mother5Other	1.00 (0.98 - 1.03)	0.739	1.00 (0.98 - 1.03)	1.01 (0.98 - 1.03)	0.607	1.01 (0.98 -1.03)
Birth_year2012:SES.mother5Other	1.00 (0.98 - 1.03)	0.824	1.00 (0.98 - 1.03)	1.00 (0.98 - 1.03)	0.841	1.00 (0.98 -1.03)
Birth_year2013:SES.mother5Other	1.00 (0.98 - 1.03)	0.757	1.00 (0.98 - 1.03)	1.01 (0.98 - 1.03)	0.639	1.00 (0.98 -1.03)
Birth_year2014:SES.mother5Other	1.00 (0.98 - 1.03)	0.848	1.00 (0.97 - 1.03)	1.00 (0.98 - 1.03)	0.871	1.00 (0.97 -1.03)
Birth_year2015:SES.mother5Other	1.01 (0.98 - 1.03)	0.710	1.00 (0.98 - 1.03)	1.00 (0.98 - 1.03)	0.774	1.00 (0.98 -1.03)
Birth_year2016:SES.mother5Other	1.02 (0.99 - 1.04)	0.293	1.01 (0.99 - 1.04)	1.01 (0.98 - 1.04)	0.488	1.01 (0.98 -1.04)
Birth_year2017:SES.mother5Other	1.01 (0.99 - 1.04)	0.338	1.01 (0.98 - 1.04)	1.01 (0.98 - 1.04)	0.519	1.01 (0.98 -1.04)
Birth_year2018:SES.mother5Other	1.01 (0.99 - 1.04)	0.332	1.01 (0.98 - 1.04)	1.02 (0.99 - 1.05)	0.318	1.01 (0.98 -1.04)
Birth_year2019:SES.mother5Other	1.03 (1.00 - 1.06)	0.056	1.03 (1.00 - 1.06)	1.03 (1.00 - 1.06)	0.064	1.03 (1.00 -1.06)
Birth_year2020:SES.mother5Other	1.05 (1.02 - 1.08)	0.003	1.04 (1.01 - 1.08)	1.05 (1.01 - 1.08)	0.005	1.04 (1.01 -1.07)
Birth_year2009:SES.mother5Unknown	NA	NA	0.99 (0.88 - 1.09)	NA	NA	1.01 (0.89 -1.12)
Birth_year2010:SES.mother5Unknown	NA	NA	1.02 (0.91 - 1.12)	NA	NA	1.06 (0.95 -1.17)
Birth_year2011:SES.mother5Unknown	NA	NA	1.02 (0.91 - 1.13)	NA	NA	1.07 (0.95 -1.18)
Birth_year2012:SES.mother5Unknown	NA	NA	1.02 (0.91 - 1.13)	NA	NA	1.08 (0.96 -1.19)
Birth_year2013:SES.mother5Unknown	NA	NA	1.06 (0.95 - 1.17)	NA	NA	1.08 (0.96 -1.19)
Birth_year2014:SES.mother5Unknown	NA	NA	1.06 (0.95 - 1.17)	NA	NA	1.08 (0.96 -1.19)
Birth_year2015:SES.mother5Unknown	NA	NA	0.98 (0.86 - 1.10)	NA	NA	1.05 (0.93 -1.17)
Birth_year2016:SES.mother5Unknown	NA	NA	1.00 (0.88 - 1.12)	NA	NA	1.03 (0.90 -1.15)
Birth_year2017:SES.mother5Unknown	NA	NA	1.01 (0.89 - 1.14)	NA	NA	1.06 (0.93 -1.19)
Birth_year2018:SES.mother5Unknown	NA	NA	1.02 (0.90 - 1.15)	NA	NA	1.03 (0.90 -1.16)
Birth_year2019:SES.mother5Unknown	NA	NA	1.03 (0.90 - 1.15)	NA	NA	1.07 (0.94 -1.20)
Birth_year2020:SES.mother5Unknown	NA	NA	0.95 (0.84 - 1.07)	NA	NA	0.86 (0.74 -0.98)
Birth_year2009:UrbanisationExtremely urbanised	1.00 (0.98 - 1.03)	0.744	1.00 (0.98 - 1.03)	1.00 (0.98 - 1.03)	0.783	1.00 (0.98 -1.03)
Birth_year2010:UrbanisationExtremely urbanised	1.00 (0.98 - 1.03)	0.761	1.00 (0.98 - 1.03)	1.01 (0.98 - 1.03)	0.490	1.01 (0.98 -1.03)
Birth_year2011:UrbanisationExtremely urbanised	1.01 (0.98 - 1.03)	0.533	1.01 (0.98 - 1.03)	1.01 (0.99 - 1.04)	0.377	1.01 (0.99 -1.03)

Birth_year2012:UrbanisationExtremely urbanised	1.01 (0.99 - 1.03)	0.445	1.01 (0.99 - 1.03)	1.01 (0.99 - 1.04)	0.305	1.01 (0.99 -1.04)
Birth_year2013:UrbanisationExtremely urbanised	1.01 (0.99 - 1.04)	0.296	1.01 (0.99 - 1.04)	1.01 (0.99 - 1.04)	0.261	1.01 (0.99 -1.04)
Birth_year2014:UrbanisationExtremely urbanised	1.01 (0.99 - 1.04)	0.307	1.01 (0.99 - 1.04)	1.01 (0.99 - 1.04)	0.270	1.01 (0.99 -1.04)
Birth_year2015:UrbanisationExtremely urbanised	1.02 (0.99 - 1.04)	0.148	1.02 (0.99 - 1.04)	1.02 (0.99 - 1.04)	0.144	1.02 (0.99 -1.04)
Birth_year2016:UrbanisationExtremely urbanised	1.02 (0.99 - 1.04)	0.121	1.02 (1.00 - 1.05)	1.02 (1.00 - 1.05)	0.084	1.02 (1.00 -1.05)
Birth_year2017:UrbanisationExtremely urbanised	1.01 (0.99 - 1.04)	0.236	1.02 (0.99 - 1.04)	1.02 (0.99 - 1.04)	0.172	1.02 (0.99 -1.04)
Birth_year2018:UrbanisationExtremely urbanised	1.02 (1.00 - 1.04)	0.114	1.02 (1.00 - 1.05)	1.02 (1.00 - 1.05)	0.070	1.02 (1.00 -1.05)
Birth_year2019:UrbanisationExtremely urbanised	1.01 (0.99 - 1.04)	0.309	1.01 (0.99 - 1.04)	1.02 (1.00 - 1.04)	0.115	1.02 (1.00 -1.05)
Birth_year2020:UrbanisationExtremely urbanised	1.01 (0.98 - 1.03)	0.517	1.01 (0.99 - 1.03)	1.01 (0.99 - 1.04)	0.367	1.01 (0.99 -1.04)
Birth_year2009:UrbanisationHardly urbanised	1.00 (0.98 - 1.03)	0.808	1.00 (0.98 - 1.03)	1.00 (0.98 - 1.03)	0.737	1.00 (0.98 -1.03)
Birth_year2010:UrbanisationHardly urbanised	1.00 (0.98 - 1.03)	0.856	1.00 (0.98 - 1.02)	1.01 (0.98 - 1.03)	0.649	1.01 (0.98 -1.03)
Birth_year2011:UrbanisationHardly urbanised	1.00 (0.98 - 1.03)	0.768	1.00 (0.98 - 1.03)	1.01 (0.98 - 1.03)	0.616	1.01 (0.98 -1.03)
Birth_year2012:UrbanisationHardly urbanised	1.00 (0.98 - 1.03)	0.783	1.00 (0.98 - 1.03)	1.01 (0.98 - 1.03)	0.600	1.01 (0.98 -1.03)
Birth_year2013:UrbanisationHardly urbanised	1.00 (0.97 - 1.02)	0.876	1.00 (0.97 - 1.02)	1.00 (0.97 - 1.02)	0.890	1.00 (0.97 -1.02)
Birth_year2014:UrbanisationHardly urbanised	1.00 (0.98 - 1.02)	0.992	1.00 (0.98 - 1.02)	1.00 (0.98 - 1.02)	0.991	1.00 (0.98 -1.02)
Birth_year2015:UrbanisationHardly urbanised	1.00 (0.97 - 1.02)	0.766	1.00 (0.97 - 1.02)	1.00 (0.97 - 1.02)	0.887	1.00 (0.98 -1.02)
Birth_year2016:UrbanisationHardly urbanised	1.00 (0.98 - 1.02)	0.998	1.00 (0.98 - 1.02)	1.00 (0.98 - 1.03)	0.874	1.00 (0.98 -1.03)
Birth_year2017:UrbanisationHardly urbanised	1.00 (0.98 - 1.02)	0.936	1.00 (0.98 - 1.02)	1.00 (0.98 - 1.03)	0.853	1.00 (0.98 -1.03)
Birth_year2018:UrbanisationHardly urbanised	1.01 (0.99 - 1.03)	0.446	1.01 (0.99 - 1.03)	1.01 (0.99 - 1.03)	0.405	1.01 (0.99 -1.03)
Birth_year2019:UrbanisationHardly urbanised	1.01 (0.98 - 1.03)	0.606	1.01 (0.98 - 1.03)	1.01 (0.99 - 1.03)	0.477	1.01 (0.99 -1.03)
Birth_year2020:UrbanisationHardly urbanised	1.01 (0.98 - 1.03)	0.655	1.01 (0.98 - 1.03)	1.01 (0.98 - 1.03)	0.546	1.01 (0.98 -1.03)
Birth_year2009:UrbanisationModerately urbanised	1.00 (0.98 - 1.02)	0.868	1.00 (0.98 - 1.02)	1.00 (0.98 - 1.02)	0.950	1.00 (0.98 -1.02)
Birth_year2010:UrbanisationModerately urbanised	1.00 (0.98 - 1.03)	0.718	1.00 (0.98 - 1.03)	1.00 (0.98 - 1.03)	0.786	1.00 (0.98 -1.03)
Birth_year2011:UrbanisationModerately urbanised	1.01 (0.98 - 1.03)	0.622	1.01 (0.98 - 1.03)	1.01 (0.98 - 1.03)	0.586	1.01 (0.98 -1.03)
Birth_year2012:UrbanisationModerately urbanised	1.01 (0.99 - 1.03)	0.492	1.01 (0.99 - 1.03)	1.01 (0.98 - 1.03)	0.533	1.01 (0.98 -1.03)
Birth_year2013:UrbanisationModerately urbanised	1.01 (0.98 - 1.03)	0.555	1.01 (0.98 - 1.03)	1.01 (0.98 - 1.03)	0.613	1.01 (0.98 -1.03)
Birth_year2014:UrbanisationModerately urbanised	1.01 (0.99 - 1.03)	0.442	1.01 (0.99 - 1.03)	1.01 (0.98 - 1.03)	0.516	1.01 (0.99 -1.03)
Birth_year2015:UrbanisationModerately urbanised	1.01 (0.99 - 1.03)	0.357	1.01 (0.99 - 1.03)	1.01 (0.98 - 1.03)	0.602	1.01 (0.98 -1.03)
Birth_year2016:UrbanisationModerately urbanised	1.01 (0.99 - 1.04)	0.264	1.01 (0.99 - 1.04)	1.01 (0.99 - 1.04)	0.311	1.01 (0.99 -1.04)
Birth_year2017:UrbanisationModerately urbanised	1.01 (0.99 - 1.03)	0.455	1.01 (0.99 - 1.03)	1.01 (0.98 - 1.03)	0.550	1.01 (0.98 -1.03)
Birth_year2018:UrbanisationModerately urbanised	1.01 (0.99 - 1.04)	0.329	1.01 (0.99 - 1.04)	1.01 (0.99 - 1.04)	0.343	1.01 (0.99 -1.04)
Birth_year2019:UrbanisationModerately urbanised	1.01 (0.98 - 1.03)	0.517	1.01 (0.98 - 1.03)	1.01 (0.98 - 1.03)	0.560	1.01 (0.98 -1.03)
Birth_year2020:UrbanisationModerately urbanised	1.01 (0.99 - 1.03)	0.386	1.01 (0.99 - 1.03)	1.01 (0.99 - 1.03)	0.390	1.01 (0.99 -1.04)
Birth_year2009:UrbanisationStrongly urbanised	1.00 (0.98 - 1.02)	0.972	1.00 (0.98 - 1.02)	1.00 (0.98 - 1.02)	0.832	1.00 (0.98 -1.02)
Birth_year2010:UrbanisationStrongly urbanised	1.00 (0.98 - 1.02)	0.885	1.00 (0.98 - 1.02)	1.00 (0.98 - 1.02)	0.924	1.00 (0.98 -1.02)
Birth_year2011:UrbanisationStrongly urbanised	1.00 (0.98 - 1.03)	0.709	1.00 (0.98 - 1.03)	1.00 (0.98 - 1.03)	0.770	1.00 (0.98 -1.03)

Birth_year2012:UrbanisationStrongly urbanised	1.01 (0.98 - 1.03)	0.645	1.01 (0.98 - 1.03)	1.00 (0.98 - 1.03)	0.704	1.00 (0.98 -1.03)
Birth_year2013:UrbanisationStrongly urbanised	1.00 (0.98 - 1.03)	0.754	1.00 (0.98 - 1.03)	1.00 (0.98 - 1.03)	0.824	1.00 (0.98 -1.02)
Birth_year2014:UrbanisationStrongly urbanised	1.01 (0.98 - 1.03)	0.553	1.01 (0.99 - 1.03)	1.00 (0.98 - 1.03)	0.791	1.00 (0.98 -1.03)
Birth_year2015:UrbanisationStrongly urbanised	1.01 (0.99 - 1.03)	0.414	1.01 (0.99 - 1.03)	1.00 (0.98 - 1.03)	0.671	1.00 (0.98 -1.03)
Birth_year2016:UrbanisationStrongly urbanised	1.01 (0.99 - 1.03)	0.401	1.01 (0.99 - 1.03)	1.01 (0.98 - 1.03)	0.526	1.01 (0.99 -1.03)
Birth_year2017:UrbanisationStrongly urbanised	1.01 (0.99 - 1.03)	0.439	1.01 (0.99 - 1.03)	1.01 (0.98 - 1.03)	0.569	1.01 (0.98 -1.03)
Birth_year2018:UrbanisationStrongly urbanised	1.02 (0.99 - 1.04)	0.150	1.02 (0.99 - 1.04)	1.01 (0.99 - 1.04)	0.219	1.01 (0.99 -1.04)
Birth_year2019:UrbanisationStrongly urbanised	1.01 (0.99 - 1.03)	0.461	1.01 (0.99 - 1.03)	1.01 (0.99 - 1.03)	0.438	1.01 (0.99 -1.03)
Birth_year2020:UrbanisationStrongly urbanised	1.01 (0.99 - 1.04)	0.307	1.01 (0.99 - 1.03)	1.01 (0.99 - 1.03)	0.439	1.01 (0.99 -1.03)
Birth_year2009:UrbanisationUnknown	NA	NA	1.15 (0.60 - 1.70)	NA	NA	1.25 (0.65 -1.86)
Birth_year2010:UrbanisationUnknown	NA	NA	1.10 (0.53 - 1.68)	NA	NA	1.08 (0.42 -1.73)
Birth_year2011:UrbanisationUnknown	NA	NA	1.32 (0.72 - 1.93)	NA	NA	1.48 (0.76 -2.19)
Birth_year2012:UrbanisationUnknown	NA	NA	1.24 (0.52 - 1.96)	NA	NA	1.92 (1.00 -2.85)
Birth_year2013:UrbanisationUnknown	NA	NA	1.46 (0.75 - 2.16)	NA	NA	1.71 (0.25 -3.17)
Birth_year2014:UrbanisationUnknown	NA	NA	1.33 (0.69 - 1.97)	NA	NA	1.54 (0.47 -2.61)
Birth_year2015:UrbanisationUnknown	NA	NA	1.18 (0.45 - 1.90)	NA	NA	0.97 (-1.04 -2.98)
Birth_year2016:UrbanisationUnknown	NA	NA	1.41 (0.87 - 1.95)	NA	NA	1.63 (0.97 -2.29)
Birth_year2017:UrbanisationUnknown	NA	NA	1.40 (0.79 - 2.02)	NA	NA	1.60 (0.68 -2.53)
Birth_year2018:UrbanisationUnknown	NA	NA	1.15 (0.53 - 1.77)	NA	NA	1.32 (0.45 -2.19)
Birth_year2019:UrbanisationUnknown	NA	NA	1.36 (0.85 - 1.87)	NA	NA	1.70 (1.07 -2.33)
Birth_year2020:UrbanisationUnknown	NA	NA	1.33 (0.87 - 1.79)	NA	NA	1.40 (0.69 -2.11)
Birth_year2009:family_size4 or more children	1.01 (0.98 - 1.04)	0.708	1.01 (0.98 - 1.03)	1.00 (0.97 - 1.03)	0.875	1.00 (0.97 -1.03)
Birth_year2010:family_size4 or more children	1.01 (0.98 - 1.04)	0.740	1.00 (0.97 - 1.03)	1.00 (0.97 - 1.04)	0.775	1.00 (0.97 -1.03)
Birth_year2011:family_size4 or more children	1.01 (0.98 - 1.04)	0.500	1.01 (0.98 - 1.04)	1.01 (0.98 - 1.04)	0.510	1.01 (0.98 -1.04)
Birth_year2012:family_size4 or more children	1.00 (0.97 - 1.03)	0.840	1.00 (0.97 - 1.03)	0.99 (0.96 - 1.02)	0.702	0.99 (0.96 -1.02)
Birth_year2013:family_size4 or more children	1.01 (0.98 - 1.04)	0.453	1.01 (0.98 - 1.04)	1.01 (0.98 - 1.04)	0.641	1.01 (0.98 -1.04)
Birth_year2014:family_size4 or more children	1.00 (0.97 - 1.03)	0.940	1.00 (0.97 - 1.03)	1.00 (0.97 - 1.03)	0.999	1.00 (0.97 -1.03)
Birth_year2015:family_size4 or more children	1.00 (0.97 - 1.03)	0.977	1.00 (0.97 - 1.03)	1.00 (0.97 - 1.03)	0.986	1.00 (0.97 -1.03)
Birth_year2016:family_size4 or more children	1.01 (0.98 - 1.04)	0.596	1.01 (0.98 - 1.04)	1.01 (0.98 - 1.04)	0.706	1.01 (0.98 -1.04)
Birth_year2017:family_size4 or more children	1.02 (0.99 - 1.05)	0.295	1.02 (0.99 - 1.05)	1.01 (0.98 - 1.04)	0.526	1.01 (0.98 -1.04)
Birth_year2018:family_size4 or more children	1.01 (0.98 - 1.04)	0.722	1.00 (0.97 - 1.03)	1.00 (0.97 - 1.03)	0.927	1.00 (0.97 -1.03)
Birth_year2019:family_size4 or more children	1.00 (0.97 - 1.04)	0.759	1.00 (0.97 - 1.03)	1.00 (0.97 - 1.03)	0.876	1.00 (0.97 -1.03)
Birth_year2020:family_size4 or more children	0.99 (0.96 - 1.02)	0.375	0.99 (0.95 - 1.02)	0.98 (0.95 - 1.01)	0.287	0.98 (0.95 -1.02)
Birth_year2009:family_sizeInstitutional	1.11 (0.87 - 1.42)	0.393	1.17 (0.98 - 1.37)	1.13 (0.88 - 1.45)	0.334	1.22 (1.01 -1.42)
Birth_year2010:family_sizeInstitutional	1.07 (0.85 - 1.35)	0.548	1.13 (0.95 - 1.31)	1.12 (0.88 - 1.42)	0.343	1.24 (1.05 -1.43)
Birth_year2011:family_sizeInstitutional	1.06 (0.83 - 1.36)	0.641	1.10 (0.92 - 1.29)	1.10 (0.85 - 1.41)	0.472	1.20 (1.00 -1.39)

Birth_year2012:family_sizeInstitutional	1.10 (0.86 - 1.41)	0.435	1.14 (0.95 - 1.33)	1.11 (0.86 - 1.43)	0.425	1.20 (1.00 -1.39)
Birth_year2013:family_sizeInstitutional	1.09 (0.86 - 1.39)	0.457	1.13 (0.94 - 1.32)	1.13 (0.89 - 1.44)	0.314	1.20 (1.01 -1.39)
Birth_year2014:family_sizeInstitutional	1.04 (0.82 - 1.32)	0.737	1.07 (0.89 - 1.26)	1.05 (0.82 - 1.34)	0.712	1.13 (0.94 -1.32)
Birth_year2015:family_sizeInstitutional	1.05 (0.82 - 1.35)	0.718	1.05 (0.86 - 1.24)	1.12 (0.87 - 1.44)	0.387	1.15 (0.95 -1.35)
Birth_year2016:family_sizeInstitutional	1.05 (0.82 - 1.35)	0.673	1.03 (0.84 - 1.22)	1.11 (0.87 - 1.42)	0.391	1.10 (0.91 -1.30)
Birth_year2017:family_sizeInstitutional	1.06 (0.82 - 1.37)	0.644	1.03 (0.83 - 1.22)	1.12 (0.87 - 1.44)	0.377	1.11 (0.91 -1.30)
Birth_year2018:family_sizeInstitutional	1.09 (0.85 - 1.39)	0.498	1.03 (0.84 - 1.21)	1.11 (0.87 - 1.42)	0.386	1.08 (0.89 -1.27)
Birth_year2019:family_sizeInstitutional	1.02 (0.79 - 1.32)	0.878	0.95 (0.76 - 1.14)	1.05 (0.81 - 1.35)	0.729	1.00 (0.80 -1.19)
Birth_year2020:family_sizeInstitutional	1.09 (0.86 - 1.40)	0.475	1.08 (0.89 - 1.27)	1.16 (0.90 - 1.48)	0.250	1.11 (0.91 -1.30)
Birth_year2009:family_sizeUnknown	NA	NA	0.92 (0.36 - 1.47)	NA	NA	0.82 (0.22 -1.43)
Birth_year2010:family_sizeUnknown	NA	NA	0.92 (0.34 - 1.50)	NA	NA	0.95 (0.29 -1.60)
Birth_year2011:family_sizeUnknown	NA	NA	0.80 (0.19 - 1.41)	NA	NA	0.71 (0.00 -1.43)
Birth_year2012:family_sizeUnknown	NA	NA	0.83 (0.10 - 1.56)	NA	NA	0.55 (-0.38 -1.47)
Birth_year2013:family_sizeUnknown	NA	NA	0.71 (0.01 - 1.42)	NA	NA	0.63 (-0.84 -2.09)
Birth_year2014:family_sizeUnknown	NA	NA	0.78 (0.13 - 1.43)	NA	NA	0.69 (-0.39 -1.77)
Birth_year2015:family_sizeUnknown	NA	NA	0.96 (0.23 - 1.69)	NA	NA	1.18 (-0.83 -3.20)
Birth_year2016:family_sizeUnknown	NA	NA	0.80 (0.25 - 1.35)	NA	NA	0.75 (0.08 -1.41)
Birth_year2017:family_sizeUnknown	NA	NA	0.80 (0.18 - 1.42)	NA	NA	0.73 (-0.20 -1.66)
Birth_year2018:family_sizeUnknown	NA	NA	0.99 (0.36 - 1.62)	NA	NA	0.95 (0.08 -1.83)
Birth_year2019:family_sizeUnknown	NA	NA	0.86 (0.34 - 1.37)	NA	NA	0.76 (0.13 -1.40)
Birth_year2020:family_sizeUnknown	NA	NA	0.86 (0.40 - 1.32)	NA	NA	1.00 (0.29 -1.71)
Birth_year2009:Daycare2No daycare	1.00 (0.98 - 1.01)	0.792	1.00 (0.98 - 1.01)	1.00 (0.98 - 1.01)	0.783	1.00 (0.98 -1.01)
Birth_year2010:Daycare2No daycare	1.00 (0.98 - 1.01)	0.564	1.00 (0.98 - 1.01)	0.99 (0.98 - 1.01)	0.469	0.99 (0.98 -1.01)
Birth_year2011:Daycare2No daycare	1.00 (0.98 - 1.01)	0.642	1.00 (0.98 - 1.01)	0.99 (0.98 - 1.01)	0.499	1.00 (0.98 -1.01)
Birth_year2012:Daycare2No daycare	0.99 (0.98 - 1.01)	0.473	1.00 (0.98 - 1.01)	0.99 (0.98 - 1.01)	0.380	0.99 (0.98 -1.01)
Birth_year2013:Daycare2No daycare	0.99 (0.97 - 1.01)	0.227	0.99 (0.98 - 1.01)	0.99 (0.98 - 1.01)	0.262	0.99 (0.98 -1.01)
Birth_year2014:Daycare2No daycare	0.98 (0.97 - 1.00)	0.054	0.99 (0.97 - 1.00)	0.98 (0.97 - 1.00)	0.051	0.98 (0.97 -1.00)
Birth_year2015:Daycare2No daycare	0.98 (0.96 - 1.00)	0.017	0.98 (0.96 - 1.00)	0.98 (0.96 - 1.00)	0.015	0.98 (0.96 -1.00)
Birth_year2016:Daycare2No daycare	0.97 (0.95 - 0.98)	<0.001	0.97 (0.95 - 0.98)	0.97 (0.95 - 0.98)	<0.001	0.97 (0.95 -0.98)
Birth_year2017:Daycare2No daycare	0.96 (0.94 - 0.98)	<0.001	0.96 (0.94 - 0.98)	0.96 (0.94 - 0.98)	<0.001	0.96 (0.94 -0.98)
Birth_year2018:Daycare2No daycare	0.95 (0.94 - 0.97)	<0.001	0.95 (0.93 - 0.97)	0.95 (0.94 - 0.97)	<0.001	0.95 (0.94 -0.97)
Birth_year2019:Daycare2No daycare	0.94 (0.92 - 0.96)	<0.001	0.94 (0.92 - 0.96)	0.94 (0.92 - 0.96)	<0.001	0.94 (0.92 -0.96)
Birth_year2020:Daycare2No daycare	0.92 (0.90 - 0.93)	<0.001	0.92 (0.90 - 0.94)	0.93 (0.91 - 0.94)	<0.001	0.93 (0.91 -0.95)

Supplementary Table S6. Adjusted relative change in DTaP-IPV vaccination coverage

	Birth cohort											
	2009	2010	2011	2012	2013	2014	2015	2016	2017	2018	2019	2020
Education level mother (ref: high)												
Not high	-0,05	-0,05	-0,21	-0,50	-0,11	-0,27	-0,65	-0,95	-0,87	-0,88	-0,76	-1,7
SDI quartiles (ref: fourth quartile)												
First	-1,45	-1,83	-1,94	-1,99	-2,54	-2,99	-4,03	-4,83	-4,65	-4,29	-4,90	-6,13
Second	-0,42	-1,36	-1,53	-1,80	-2,17	-2,85	-3,67	-4,44	-3,78	-4,27	-4,09	-4,67
Third	0,08	-0,51	-0,58	-0,96	-0,96	-1,60	-2,41	-2,70	-2,15	-2,05	-1,85	-2,08
Income source mother (ref: job in employment)												
Self-employed	-3,35	-3,79	-4,09	-4,75	-5,21	-6,17	-6,76	-7,43	-5,94	-6,39	-6,23	-7,39
Benefit recipient	-0,83	-0,50	-1,00	-0,63	-0,70	-1,57	-2,84	-2,1	-1,36	-0,73	-0,31	-1,21
Recipient benefit	0,19	-0,90	-2,25	-2,99	-2,76	-3,58	-5,19	-4,21	-3,49	-3,01	-0,63	-5,74
Student	-2,55	-1,95	-2,42	-3,10	-1,67	-3,55	-5,95	-5,80	-3,64	-4,53	-4,50	-6,02
Other	-5,40	-5,54	-5,23	-5,81	-5,40	-6,23	-6,37	-5,97	-5,60	-5,22	-3,70	-2,31
Country of origin (ref: The Netherlands)												
Europe excl NL	-1,32	-1,09	-1,34	-1,67	-1,59	-1,99	-2,19	-2,52	-2,22	-2,43	-2,06	-2,44
Indonesia	0,88	0,58	-0,34	0,16	0,02	-0,36	-1,15	-1,18	-0,52	-1,00	-0,82	-2,16
Morocco	3,85	4,36	3,77	3,64	2,70	1,17	-2,81	-5,74	-6,26	-9,18	-15,03	-22,83
Other, Africa	-0,73	0,48	2,11	1,37	1,74	1,01	1,50	1,15	1,73	1,51	1,03	0,57
Other, America/Oceania	-2,54	-2,08	-1,78	-2,97	-2,04	-2,31	-2,55	-2,17	-2,23	-1,65	-2,01	-2,47
Other, Asia	0,52	1,22	1,48	1,43	1,58	0,76	-0,71	0,71	2,39	2,27	2,69	2,55
Suriname	0,58	0,82	0,84	0,92	0,81	0,65	-0,15	-2,10	-0,80	-2,46	-3,34	-5,54
The Dutch Caribbean	0,83	0,08	0,25	-0,14	0,78	0,48	-0,32	-2,44	-1,75	-3,46	-4,50	-6,39
Turkey	3,54	3,89	3,62	3,81	3,83	3,15	1,99	-0,02	-0,58	-1,49	-5,03	-9,07
Level of urbanization (ref: not urbanized)												
Extremely	0,52	0,68	0,68	0,66	0,84	0,34	0,47	0,63	0,62	1,03	1,02	0,01
Strongly	1,35	1,34	1,33	1,22	1,10	0,68	0,54	0,61	0,97	1,59	1,35	1,18
Moderately	1,13	1,16	1,25	1,14	1,07	0,77	0,29	0,71	0,65	0,92	0,77	0,93

Hardly	1,26	1,05	0,87	0,70	-0,03	-0,35	-0,83	-0,64	-0,17	0,46	0,61	0,30
Family size (ref: 1-3 children)												
≥ 4 children	-8,35	-8,49	-8,18	-9,84	-8,58	-9,66	-9,98	-9,58	-8,83	-9,72	-9,37	-11,26
Institutional	-2,65	-3,86	-6,27	-5,44	-3,34	-11,01	-5,28	-5,95	-4,86	-5,51	-11,0	-1,90
Day-care attendance (ref: day-care yes)												
Day-care no	-2,32	-3,01	-3,20	-3,56	-3,71	-4,81	-5,55	-6,84	-7,27	-7,92	-8,87	-10,39

Supplementary Table S7. Distribution of sociodemographic groups and number of unvaccinated children in birth cohort 2020

Category		Population (N)	Total population (%)	Unvaccinated N	Unvaccinated population (%)
Total		169500	100%	17990	100%
Education level mother					
	High	73790	43,5%	5000	27,8%
	Not high	95710	56,5%	12990	72,2%
Income household					
	First quartile	18500	10,9%	3420	19,0%
	Second quartile	44860	26,5%	6100	33,9%
	Third quartile	60590	35,7%	4790	26,6%
	Fourth quartile	43400	25,6%	2750	15,3%
Income source mother					
	Job in employment	121260	71,5%	8910	49,5%
	Self-employed	13660	8,1%	2090	11,6%
	Benefit recipient	18510	10,9%	3380	18,8%
	Recipient benefit	270	0,2%	50	0,3%
	Student	2450	1,4%	390	2,2%
	Other	11750	6,9%	2420	13,5%
Country of origin					
	The Netherlands	97260	57,4%	7230	40,2%
	Europe (excl. The Netherlands)	20650	12,2%	2540	14,1%
	Morocco	8020	4,7%	2770	15,4%
	Turkey	6530	3,9%	1350	7,5%
	Suriname	5500	3,2%	790	4,4%
	The Dutch Caribbean	3720	2,2%	610	3,4%
	Indonesia	4270	2,5%	340	1,9%

Other, America/Oceania	5090	3,0%	500	2,8%
Other, Africa	6790	4,0%	880	4,9%
Other, Asia	11670	6,9%	980	5,4%
Level of urbanisation				
Extremely urbanised	38100	22,5%	4990	27,7%
Strongly urbanised	42400	25,0%	4110	22,8%
Moderately urbanised	31940	18,8%	2810	15,6%
Hardly urbanised	28430	16,8%	2400	13,3%
Not urbanised	25940	15,3%	2650	14,7%
Family-size				
1-3 children	155820	91,9%	14230	79,1%
≥ 4 children	10740	6,3%	2630	14,6%
Institutional household	380	0,2%	120	0,7%
Day care attendance				
Yes	123520	72,9%	8000	44,5%
No	45980	27,1%	9990	55,5%
